# The Effects of Combined Exposure to Simulated Microgravity, Ionizing Radiation, and Cortisol on the *In Vitro* Wound Healing Process

**DOI:** 10.3390/cells12020246

**Published:** 2023-01-07

**Authors:** Wilhelmina E. Radstake, Kiran Gautam, Silvana Miranda, Randy Vermeesen, Kevin Tabury, Emil Rehnberg, Jasmine Buset, Ann Janssen, Liselotte Leysen, Mieke Neefs, Mieke Verslegers, Jürgen Claesen, Marc-Jan van Goethem, Uli Weber, Claudia Fournier, Alessio Parisi, Sytze Brandenburg, Marco Durante, Bjorn Baselet, Sarah Baatout

**Affiliations:** 1 Radiobiology Unit, Belgian Nuclear Research Centre (SCK CEN), 2400 Mol, Belgium; 2 Department of Molecular Biotechnology, Faculty of Bioscience Engineering, Ghent University, 9000 Ghent, Belgium; 3 Department of Biomedical Engineering, University of South Carolina, Columbia, SC 29208, USA; 4 Microbiology Unit, Belgian Nuclear Research Centre (SCK CEN), 2400 Mol, Belgium; 5 Department of Epidemiology and Data Science, Amsterdam Universitair Medische Centra, VU University, 1081 HV Amsterdam, The Netherlands; 6 Department of Radiation Oncology, University Medical Center Groningen, University of Groningen, 9712 CP Groningen, The Netherlands; 7 GSI Helmholtzzentrum für Schwerionenforschung GmbH, Biophysics Division, 64291 Darmstadt, Germany; 8 Radiation Protection Dosimetry and Calibration Expert Group, Belgian Nuclear Research Centre (SCK CEN), 2400 Mol, Belgium; 9 Department of Radiation Oncology, Mayo Clinic, Jacksonville, FL 32224, USA; 10 Condensed Matter Physics, Technische Universität Darmstadt, 64289 Darmstadt, Germany

**Keywords:** simulated microgravity, ionizing radiation, iron ions, carbon ions, protons, cortisol, fibroblast, *in vitro*, wound healing

## Abstract

Human spaceflight is associated with several health-related issues as a result of long-term exposure to microgravity, ionizing radiation, and higher levels of psychological stress. Frequent reported skin problems in space include rashes, itches, and a delayed wound healing. Access to space is restricted by financial and logistical issues; as a consequence, experimental sample sizes are often small, which limits the generalization of the results. Earth-based simulation models can be used to investigate cellular responses as a result of exposure to certain spaceflight stressors. Here, we describe the development of an *in vitro* model of the simulated spaceflight environment, which we used to investigate the combined effect of simulated microgravity using the random positioning machine (RPM), ionizing radiation, and stress hormones on the wound-healing capacity of human dermal fibroblasts. Fibroblasts were exposed to cortisol, after which they were irradiated with different radiation qualities (including X-rays, protons, carbon ions, and iron ions) followed by exposure to simulated microgravity using a random positioning machine (RPM). Data related to the inflammatory, proliferation, and remodeling phase of wound healing has been collected. Results show that spaceflight stressors can interfere with the wound healing process at any phase. Moreover, several interactions between the different spaceflight stressors were found. This highlights the complexity that needs to be taken into account when studying the effect of spaceflight stressors on certain biological processes and for the aim of countermeasures development.

## 1. Introduction

Human space exploration is expected to increase in the coming decades with a rise in both commercial and touristic spaceflight as well as deep-space interplanetary exploration missions. However, there are many health risks associated with long-term spaceflight. One of the most frequently reported issues by astronauts during their space missions are related to skin sensitivity (1.12/flight year, compared to 0.97/flight year for other notable medical events, such as upper respiratory symptoms [1]) and include itches and dryness of the skin, as well as rashes and delayed wound healing [1,2,3]. Small cutaneous injuries and delayed healing of wounds, with pus-forming wounds reported on wrists, fingers, and feet, were observed during spaceflight [1]. Delayed wound healing is a threat for astronauts’ health as wounds disrupt the anatomy of the skin, thereby compromising its barrier function, which forms an active protection from environmental hazards [4]. The skin heals wounds through a multiphase and multicomponent process. After wounding, a blood clot forms, which stops the bleeding. During the inflammatory phase, immune cells migrate to the wound site to clear any pathogens and debris. Cytokines and growth factors expressed by several cell types, including fibroblast, act as chemoattractants and stimulate the migration of different cell types [5,6,7]. During the next phase, fibroblasts, keratinocytes, and endothelial cells migrate to the wound and start proliferating, which marks the next phase: the proliferating phase. The migration of these cells requires the continuous remodeling of several cytoskeletal components, whose function is to provide the cell with adhesion, contractility, and polarity [8,9]. Finally, during the remodeling phase, the wound will further contract and fibroblasts excrete an extracellular matrix (ECM) to remodel the tissue and form a scar. This final phase can last for many weeks after injury [9,10]. Interference in the wound healing process at any phase can lead to a delayed or defective repair and increases the risk of infection and health complications.

In space, astronauts are exposed to an environment different from the one on Earth. Long-term microgravity exposure deconditions the weight-bearing musculoskeletal system as well as the cardiovascular system. It induces upward-fluid-shifts, which may be linked to ocular and visual acuity problems that are clinically classified as spaceflight associated neuro-ocular syndrome (SANS) [11,12,13,14,15]. Furthermore, higher levels of ionizing radiation increase the risks for developing cataracts, cardiovascular diseases, as well as the development of cancers later in life [16,17,18,19,20,21]. Finally, the confined and isolated environment of the spacecraft together with high workload increases the levels of psychological stress, and elevated stress hormone levels have been measured after both short- and long-term spaceflight [22,23,24,25,26]. Long-term exposure to this spaceflight environment has shown to induce changes in astronauts’ skin (as reviewed in Radstake et al. [27]), including a delayed epidermal proliferation, loss of elasticity, and degradation of dermal fibers [28]. Besides, erythema and skin sensitivity in gravity-dependent areas have been reported post-flight in one astronaut who stayed in space for almost one year [29]. Furthermore, thinning of the epidermis and decreased melanin concentration have been observed [30]. On the contrary, improvement of hydration and barrier function as well as an unchanged skin density and thickness have also been reported [31]. This indicated the variety in individual skin responses during spaceflight and highlights the need to further investigate skin responses to the spaceflight environment with larger sample sizes.

Unfortunately, access to astronaut samples is limited and in orbit experiments are costly and restricted due to logistical issues. To overcome these issues, *in vivo* and *in vitro* simulation models can be used to mimic certain aspects of the spaceflight environment and investigate their effects on different physiological systems. To simulate the effects of microgravity on cells, several ground-based facilities are available, such as clinostats, the RPM, and magnetic levitation [32]. The RPM rotates with random velocity and direction around three axes, thereby averaging the total gravity vector experienced by the cells to values below 0.003*g* [33,34]. To mimic certain aspects of radiation encountered in space, high-energy ion beams obtained at accelerator facilities can provide insights into the biological effect of exposure to cosmic radiation [35]. Finally, biological effects related to chronic stress can be investigated by inducing elevated levels of glucocorticoids. *In vitro*, this is often achieved by administrating soluble glucocorticoids to the cell culture medium.

As reviewed in Radstake et al. [27], each of these spaceflight stressors alone can influence the proper functioning of cells and harm the integrity of the skin. Microgravity can affect the mechanosensitive structures of the cell, thereby inducing changes in the cells’ morphology and function. Gravitational unloading during spaceflight results in numerous cell-type-dependent alterations including alterations in cell proliferation, differentiation, expression of signaling molecules as well as gene expression [36,37]. Exposure to ionizing radiation can affect the function of fibroblasts by inducing DNA damage, apoptosis, and inflammation, and by altering gene expression, cell proliferation, and differentiation [38,39,40,41,42,43]. Finally, the effect of glucocorticoids on cells is regulated by the glucocorticoid receptor, which is a ligand-dependent transcription factor. Different isoforms of the glucocorticoid receptor differently regulate gene transcription, and therefore, the cellular response to glucocorticoids can vary largely among individuals, but also within different tissues at different phases of the cell cycle [44,45].

It is still poorly understood how these spaceflight stressors may interact and how such interactions may affect the skin. Furthermore, only a handful of studies have examined how the spaceflight environment affects the complex multi-phase process of cutaneous wound healing [46,47,48,49]. To address these issues, this work investigates the effect of combined exposure to simulated microgravity, ionizing radiation, and psychological stress on fibroblasts’ function related to the wound healing process. To this aim, we used an *in vitro* wound healing assay of human primary dermal fibroblasts to study the expression of inflammatory cytokines and growth factors, relevant for the inflammatory phase of wound healing. Next, migration capacity and cytoskeletal reorganization, which are crucial for the proliferation phase, as well as expression of the dermal matrix proteins needed to remodel the skin tissue after wounding, were investigated.

## 2. Materials and Methods

### 2.1. Fibroblast Culture

Primary normal human dermal fibroblasts (NHDF) obtained from one donor (33-year-old Caucasian female) were purchased from PromoCell (C-12302) and cultured in Dulbecco’s Modified Eagle Medium containing GlutaMAX^TM^ (DMEM, GIBCO, 10566016, Thermo Fisher Scientific, Waltham, MA, USA) supplemented with 10% fetal bovine serum (FBS, GIBCO, 10500064, Fisher Scientific, Göteborg, Sweden) and 0.25% Penicillin-Streptomycin (Pen-Strep, Sigma-Aldrich, P4333, Merck KGaA, Darmstadt, Germany). Cells were incubated at 37 °C in a humidified atmosphere containing 5% CO_2_. Cells were passaged at 80–90% confluence using 0.05% Trypsin-EDTA (GIBCO, 25300062, Thermo Fisher Scientific, Waltham, MA, USA). All experiments were performed with asynchronized cells and at room temperature.

### 2.2. Experimental Procedures

**Figure 1:**
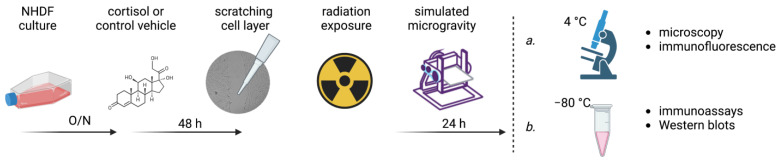
Schematic overview of experimental procedure. During each experiment, cells from the same passage number were seeded and left to attach overnight (O/N). The following day, cells were exposed to either cortisol (1 µmol/L) or a control vehicle which was diluted in the culture medium. After 48 h of incubation, cell monolayers were scratched and then irradiated. Irradiation at doses of 0.1, 0.5, or 1 Gy (or sham-irradiated controls) were performed using X-rays, protons, carbon ions, or iron ions. After irradiation, cells were placed in a simulated microgravity environment (or 1*g* control) using the random positioning machine for 24 h. Afterwards, cells were either fixed and stored at 4 °C for further processing for (immunofluorescence) microscopy to evaluate wound closure and cytoskeletal rearrangements (**a**), or cell lysates were collected and stored at −80 °C to be further processed for immunoassays to investigate the expression of cytokines and growth factors and Western blot assays for ECM protein expression (**b**). All steps were performed under similar conditions during each experiment and during processing afterwards, including distribution of samples within the well-plates and gels.

Figure 1 shows a schematic representation of the experimental procedures. Cells were seeded inside SlideFlasks (Thermo Scientific Nunc Lab-Tek, 170920) or T12.5 flasks (Corning) at densities of ~10,000 cells/cm^2^ using full serum DMEM and left to attach overnight. For each condition, a total of six replicates were used. Cells were then washed with phosphate-buffered saline (PBS) and incubated with DMEM containing hydrocortisone (HC, Sigma Aldrich, H0888, Merck KGaA, Darmstadt, Germany) at a concentration of 1 µmol/L or a control vehicle. This concentration was chosen as they are representative of circulating cortisol levels during sustained stress conditions and similar levels of cortisol have been measured in astronauts returning form space [22,25,50,51]. The hydrocortisone was first dissolved in 96% ethanol at a concentration of 1 mg/mL and further diluted in PBS to obtain a stock solution of 100 µmol/L hydrocortisone. The control vehicle consisted of 96% ethanol dissolved in PBS. Both solutions were then 1/100 diluted in the cell culture media. After 48 h of exposure to HC, cells were scratched using a bent 1 mL pipette tip and washed twice with PBS. Cell holders were then completely filled with CO_2_-calibrated media containing either HC or the control vehicle, and airtight sealed using polymer integrated caps. Next, cells were irradiated with either X-rays, protons, carbon ions, or iron ions (see Section 2.3 for an overview of different radiation facilities and parameters) at doses of 0.1, 0.5, and 1 Gy, to compare between relatively low space-relevant doses [52] and a higher dose to test for dose-specific effects, or sham-irradiated (an overview of irradiation parameters is provided in Table 1). After irradiation, cells were placed on an RPM or in a 1*g* environment as controls for 24 h. Flasks that developed air bubbles while they were placed on the RPM were discarded. Afterwards, cells in SlideFlasks were rinsed with PBS and fixed using 10% Formalin solution (Sigma Aldrich, HT5014, Merck KGaA, Darmstadt, Germany).

Cells grown in T12.5 flasks were used for collection of cell lysates. In short, cells were dissociated using 0.25% Trypsin-EDTA (GIBCO, 25200072, Thermo Fisher Scientific, Waltham, MA, USA) and pellets were collected and washed using ice-cold PBS, after which they were re-suspended in ice-cold radioimmunopreciptation assay (RIPA) buffer (Pierce, 89901, Thermo Fisher Scientific, Waltham, MA, USA) containing Halt protease and phosphatase inhibitor cocktail (Thermo Scientific, 78440). Tissue Lyser II (Qiagen) at 30.00/s was used for 2 min, after which cells lysates were centrifuged at 14,000× *g* for 10 min at 4 °C to pellet down the cell debris and supernatant was collected. Cell lysates were stored at −80 °C.

### 2.3. Exposure to Ionizing Radiation

Fibroblasts were exposed to several radiation qualities including photons, protons, carbon ions, and iron ions at different radiation facilities in Europe. An overview of the radiation qualities, mean linear energy transfer (LET) values, and the microdosimetric quantities for the different radiation exposures can be found in Table 1. The quantities listed in Table 1 were obtained by means of computer simulations (see Supplementary Materials) with PHITS [53].

Cells were exposed to H250 X-rays at the Laboratory of Nuclear Calibration of the Belgian Nuclear Research Centre (SCK CEN) in Mol, Belgium. Flasks were placed on a Plexiglass plate in a horizontal position and irradiated with H250 X-rays from the top at 50 cm distance. The photon energy spectrum of the H250 X-rays can be found in the Supplementary Materials. The exposures to carbon ions (90 MeV/n) and protons (150 MeV) were carried out at the Particle Therapy Research Center (PARTEC) facility in Groningen, The Netherlands. Samples were irradiated through the bottom of the culture vessel positioned vertically in the plateau of the Bragg curve. Irradiations were performed with a scanned beam with a homogeneous fluence. During proton irradiation, dose build-up was achieved using an 18 mm polycarbonate plate. Dose rates of approximately 0.5 Gy/min were used. Finally, fibroblasts were exposed to 1 GeV/n iron ions at the GSI-FAIR facility in Darmstadt, Germany. Samples were irradiated through the bottom of the culture vessel positioned in a vertical position with a scanned pencil beam with a homogeneous fluence in the plateau of the Bragg curve. For iron ions, a dose of 1 Gy corresponds to a fluence of 4 × 10^6^ ions/cm^2^.

### 2.4. Exposure to Simulated Microgravity

After irradiation, cells were placed on the RPM to expose them to simulated microgravity. Cell culture flasks were completely filled with medium and airtight sealed using caps filled with a polymer (SYLGARD 184 Silicon Elastomer, Dow, 01673921). Cells were exposed to simulated microgravity for 24 h. To minimize the effects of parasitic accelerations and shear stress acting upon the cells, moderate velocity of average 60 deg/s was chosen. In addition, cells were placed within 10 cm from the center of rotation.

### 2.5. Cytokine and Growth-Factor Synthesis

Multiplex immunoassays (LXSAHM-03, R&D Systems, Mineapolis, MN, USA) were used to investigate fibroblast expression levels of interleukin-6 (IL-6), interleukin-1 receptor antagonist (IL-1RA), and platelet-derived growth factor alpha (PDGF-α) in cell lysates following the manufacturer’s instructions. The Luminex MAGPIX system with xPONENT 4.3 software (Luminex Corporation, Austin, TX, USA. A DiaSorin Company) instrument was used for analyses of the multiplex immunoassays (using Belysa ^®^ Immunoassay Curve Fitting Software (Merck KGaA, Darmstadt, Germany)). The ELISA assay (Human TGF beta 1 Elisa kit, ab108912, Abcam, Cambridge, UK) was further used for determining levels of transforming growth factor beta 1 (TGF-β1). Absorbance at 450 nm was measured using a 96 Plate Reader (Enzo Life Sciences, Inc., East Farmingdale, NY, USA) and additional background subtraction was done at 570 nm (analysis with Byonoy Software, Byonoy GmbH). Total protein quantification was done with a bicinchoninic acid assay (Sigma Aldrich, Merck KGaA, Darmstadt, Germany) and these data were used for normalization of the data.

### 2.6. In Vitro Scratch Wound Assay

Confluent cell monolayers were scratched after 48 h of cortisol incubation using a 1 mL bended pipette tip. Afterwards, the scratch was observed under a Leica microscope with a 5× objective and baseline images were captured at three different locations per scratch. To limit the effect of proliferation on wound closure, cells were incubated with medium containing 1% FBS after scratching. After fixation, cells were again imaged at the same location. Images were analyzed to determine open wound area using Matlab (R2021b, The Mathworks Inc., Natick, MA, USA). A high-throughput microscopy wound healing tool [54] was used for generating a mask threshold. Masked images were then inspected and faulty masks were excluded from the data. Relative wound closure was calculated using the following equation:

(1) $$\frac{t\left(0\right)-t\left(24\right)}{t\left(0\right)}*100\;\%$$

where *t*(0) is the pixel count in the wound area directly after scratching and *t*(24) is the pixel count in the wound area 24 h after irradiation.

Finally, obtained values were then normalized to the 1*g*, 0 Gy controls without cortisol to obtain relative migration measures.

### 2.7. Cyotskeletal Remodeling

For each condition, three out of six fixed scratched samples were used for immunocytochemical visualization of actin stress fibers and vinculin focal adhesion complexes. Fixed cells were incubated in PBS containing 0.1% Triton-X 100 and 3% bovine serum albumin (BSA, A2153n Sigma-Aldrich, Merck KGaA, Darmstadt, Germany) for 5 min at room temperature, after which they were washed with Tris-buffered saline containing 0.1% Tween (TBS-T). For blocking, a solution of 5% goat serum in Tris-NaCl-blocking buffer (TNB) was used for one hour at room temperature. Primary antibody (mouse monoclonal anti-vinculin (7f9), Santa Cruz, sc-73614) at dilution of 1/500 in TNB was incubated overnight at 4 °C. Samples were washed with TBS and incubated with TNB containing secondary antibody (Alexa Fluor goat-anti-mouse 488, Invitrogen A11001, 1/500) and Phalloidin 594 TRITC (Invitrogen A12381) for actin stress fibers. Slides were mounted with mounting medium containing DAPI (Molecular Probes Prolong Diamond Antifade Mountant, p36962). A Nikon Ti Eclipse inverted widefield fluorescence microscope (Nikon Instruments) with a 60× objective and immersion oil and connected to a Prime BSI sCMOS camera was used to visualize the cellular components of isolated cells that had migrated into the open wound area. Z-stacks of 11 images were taken at 0.5 µm apart.

#### Image Analysis

Fiji (v1.53C, https://fiji.sc) was used for image processing. All images were summed across the *z*-axis.

**Focal adhesions:** Preprocessing steps for images of vinculin included background subtraction with rolling ball radius of 10 pixels. Afterwards, images were further processed using a median filter (radius of 6 pixels) to suppress somatic vinculin signal followed by a top-hat filter (using a disk with radius 6). Images were then thresholded following the Otsu algorithm. Finally, to determine the number of focal adhesions per images, spots larger than 75 pixels were counted per image. This threshold was determined to select mature focal adhesions that are associated with the cytoskeleton [55].

**Actin area and number of stress fibers:** Actin images were thresholded following the Triangle algorithm, after which the total area per image was measured. For determination of the number of actin stress fibers, actin images were first background subtracted with a rolling ball with a radius of 10 pixels followed by a fast Fourier transformation (FFT) and low-frequency filtering, after which an inverse FFT was applied followed by Gaussian filter (σ = 1 pixel). Finally, images were thresholded following the Otsu algorithm. Per image, the total number of stress fibers was determined by identifying particles larger than 1000 pixels with circularity between 0 and 0.1.

**Nuclei:** Per image (224 × 224 micron), the total number of nuclei was determined by thresholding DAPI signal images following the Triangle algorithm and watershed to separate bordering nuclei.

The number of nuclei was used to determine the total actin area and actin stress fibers per cell as well as the amount of vinculin focal adhesion spots per cell. These values were then normalized to the 1*g*, 0 Gy controls without cortisol to obtain measures of relative cell dimension, relative number of focal adhesions, and relative number of stress fibers per cell. Per condition, on average 170 nuclei were imaged.

### 2.8. Extracellular Matrix Protein Expression

Western blot assays were performed to determine the expression of extracellular matrix (ECM) proteins of procollagen type I as well as fibronectin. Lysates were collected as described in Section 2.2. Total protein quantification was done with bicinchoninic acid assay (Sigma Aldrich, BCA1, Merck KGaA, Darmstadt, Germany). MilliQ was used to dilute cell pellets and obtain a concentration of 0.33 µg/µL of protein per sample. A 4× Laemli buffer (1610747, BIO-RAD Laboratories) with β-mercaptoethanol (1/10) was added to the protein samples, followed by incubation at 95 °C for 10 min for samples used to measure expression of fibronectin. Per lane, 5 µg of protein samples were loaded onto a 4–15% Criterion TGX Stain-Free Precast Gel (5671085, BIO-RAD Laboratories) and transferred to a nitrocellulose membrane. Blocking was performed in 5% milk powder in TBS-T for 2 h at room temperature. Primary antibodies diluted in blocking buffer (rabbit polyclonal to collagen type I (1/1000, Abcam, ab34710) for collagen type I, and mouse monoclonal anti-fibronectin (1/1000, Sigma-Aldrich, F0916) for fibronectin) were incubated overnight at 4 °C. Additionally, primary antibodies for mouse monoclonal to glyceraldehyde 3-phosphate dehydrogenase (GAPDH) were added (1/10,000, Abcam, ab8425), which served as loading control. Membranes were then washed with TBS-T and incubated with secondary horseradish peroxidase (HRP)-conjugated antibodies diluted in blocking buffer (1/2000 for collagen type I, 1/1000 for fibronectin and 1/1000 for GAPDH). Enhanced chemiluminescence was used for the detection of HRP conjugates (1705061, BIO-RAD clarity kit used according to the manufacturer’s instructions). Blots were imaged using the Fusion FX (Vilber).

Protein bands for the target proteins were measured using the Bio1D (v15.06, Vilber Lourmat). Each band was normalized to the loading and internal control. Measures were then normalized to the 1*g*, 0 Gy controls without cortisol to obtain relative measures.

### 2.9. Statistical Analyses

General linear regression (assuming a Gaussian distribution) and data plotting was performed in R version 4.0.1 [56] to test for the main and interaction effects of the independent variables stress, gravity, dose, and radiation quality. An overview of these factors can be found in Table 2. For each experimental condition, the data were normalized to the 1*g*, 0 Gy, control without cortisol to obtain relative values. The data were checked for outliers; data points 1.5 times the interquartile range above the upper quartile and below the lower quartile were removed from the data. Models were then tested for significant interaction effects of independent variables. If interaction effects were not significant, they were removed from the model. Akaike information criterion (AIC) values were considered as measure of the goodness-of-fit of the model. The residual errors were taken into account to guarantee unbiased estimates.

## 3. Results

### 3.1. Effect of Simulated Spaceflight Environment on Synthesis of Cytokines and Growth Factors

During the initial phase of wound healing, that is the inflammatory phase, fibroblasts express a combination of different pro- and anti-inflammatory cytokines and growth factors. These cytokines and growth factors act as chemoattractants for other cells and stimulate proliferation and extracellular matrix protein expression [5,6,7]. Here, the expression of interleukin 1 receptor antagonist (IL-1RA), interleukin 6 (IL-6), platelet-derived growth factor-alpha (PDGF-α), and transforming growth factor-beta (TGF-β) in fibroblasts after exposure to simulated spaceflight stressors was tested. For each endpoint, a regression analysis was performed, which are described in more detail in the next section.

#### 3.1.1. IL-1RA Expression

IL-1RA regulates the effect of pro-inflammatory cytokine interleukin 1-alpha through competitive binding to the cells’ interleukin-1 receptor. Its upregulation during the inflammatory phase of wound healing is crucial for proper healing, and imbalance in the expression of IL-1RA leads to delayed wound healing [57].

One outlier (relative expression > 2) was identified and removed from the data. The regression model was fitted with a four-way interaction between the factors stress, gravity, dose, and radiation quality. The model was statistically significant (AIC = −1130, R^2^ = 0.5, F (63, 305) = 5.87, *p* < 0.0001). The regression coefficients for each significant predictor can be found in Supplementary Materials Table S1. Table 3 shows the analysis of variance table of the regression model for IL-1RA.

**Figure 2:**
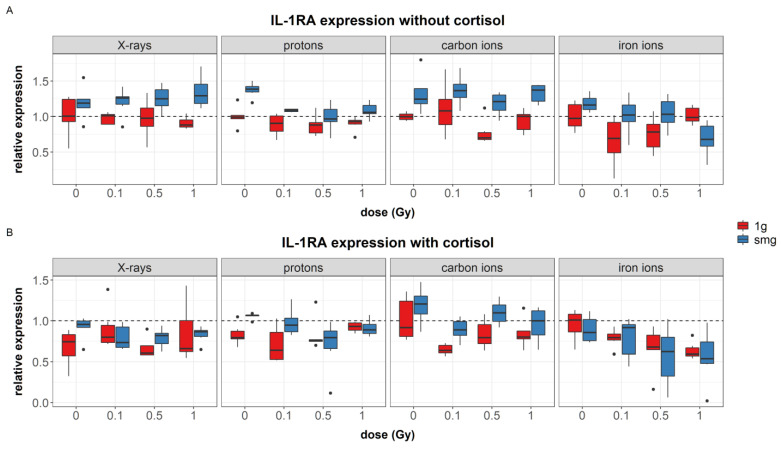
Overview of IL-1RA synthesis measured in cell lysates after exposure of NHDF to simulated spaceflight stressors as explained in Section 2.2. (**A**): Effect of simulated microgravity, dose, and radiation quality on expression of IL-1RA; (**B**): effect of simulated microgravity, dose, and radiation quality on expression of IL-1RA in cells exposed to cortisol (1 µmol/L). Measured using multiplex immunoassay. Expression values were normalized to total protein content, and divided by the average of 0 Gy, 1*g* controls to obtain relative expression values. Normalized values of 0 Gy, 1*g* controls during X-rays = 644 pg/µg, protons = 188 pg/µg, carbon ions = 325 pg/µg, iron ions = 405 pg/µg. smg = simulated microgravity, dotted line = average expression of 0 Gy, 1*g*, without cortisol. Plot shows boxplot with median as center line, box limits are upper and lower quartiles, whisker are 1.5× interquartile range, and points are group outliers. Six replicates per condition.

Cortisol exposure was found as a significant predictor of IL-1RA expression (*p* = 0.009); cells that were incubated with cortisol showed reduced expression levels of IL-1RA (Figure 2B) compared to unexposed cells (Figure 2A). Significant effects of dose were found in cells exposed to 1 Gy of irons ions only in combination with either cortisol exposure, simulated microgravity exposure, or both. This is observed in fibroblasts exposed to both cortisol and 1 Gy of iron ions, where a significant decrease in IL-1RA expression was found compared to 0 Gy groups. Furthermore, for most groups, exposure to simulated microgravity without cortisol (Figure 2A) seemed to increase the expression of IL-1RA. When simulated microgravity was combined with 1 Gy of iron ions, a significant downregulation of the expression of IL-1RA was observed.

#### 3.1.2. IL-6 Expression

IL-6 is a pro-inflammatory cytokine which plays an important role during the inflammatory phase of wound healing. A lack of expression of this cytokine has been linked to a delayed wound healing process [58].

Five outliers (relative expression > 4) were identified and removed from the data. The regression model was fitted with a four-way interaction between the factors stress, simulated microgravity, dose, and radiation quality. The model was significant (AIC = −569, R^2^ = 0.63, F (63, 261) = 7.13, *p* < 0.0001). The regression coefficients for each significant predictor can be found in Supplementary Materials Table S2. Table 4 shows the analysis of variance table of the regression model for IL-6.

**Figure 3:**
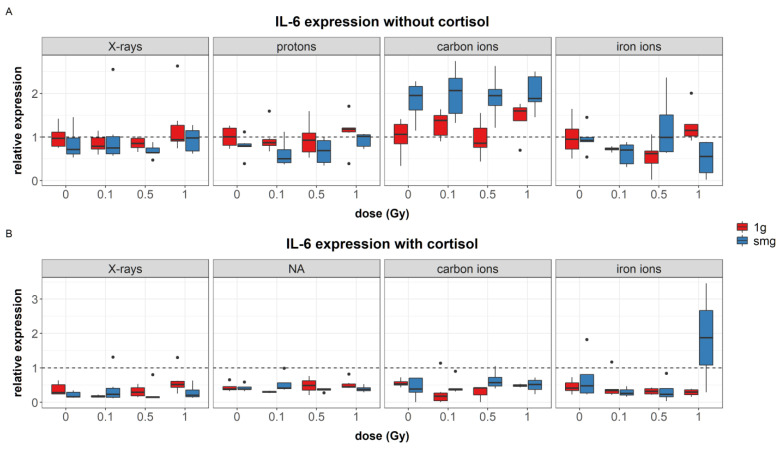
Overview of IL-6 synthesis measured in cell lysates after exposure of NHDF to simulated spaceflight stressors as explained in Section 2.2. (**A**): Effect of simulated microgravity, dose, and radiation quality on expression of IL-6; (**B**): effect of simulated microgravity, dose, and radiation quality on expression of IL-6 in cells exposed to cortisol (1 µmol/L). Measured using multiplex immunoassay. Expression values were normalized to total protein content, and divided by the average of 0 Gy, 1*g* control to obtain relative expression values. Normalized values of 0 Gy, 1*g* controls during X-rays = 3.2 pg/µg, protons = 1.2 pg/µg, carbon ions = 1.3 pg/µg, iron ions = 2.0 pg/µg. smg = simulated microgravity, dotted line = average expression of 0 Gy, 1*g*, without cortisol. Plot shows boxplot with median as center line, box limits are upper and lower quartiles, whisker are 1.5× interquartile range, and points are group outliers. Six replicates per condition.

Cortisol exposure was found as a significant predictor of IL-6 expression (*p* = 0.007) as cortisol exposure reduced fibroblasts expression of IL-6 compared to unexposed cells (Figure 3B). During carbon irradiation experiments, exposure to simulated microgravity significantly upregulated the expression of IL-6 compared to 1*g* controls (Figure 3A). However, when cells were exposed to cortisol and simulated microgravity, this effect was not observed (Figure 3B). The radiation dose only affected the expression of IL-6 in fibroblasts exposed to 1 Gy of iron ions if cells were also exposed to cortisol in combination with simulated microgravity (Figure 3B).

#### 3.1.3. PDGF-α

PDGF-α is expressed early during the inflammatory phase of wound healing. This growth factor stimulates fibroblasts proliferation and differentiation into myofibroblasts. An imbalance in the expression of PDGF-α can negatively affect the healing process (as reviewed in Werner and Grose [7]).

Because multiple readings were below detection level, relative expression levels could not be calculated, and hence, concentration levels are shown. For the same reason, no effect of dose or radiation quality could be detected and only effects of gravity and cortisol were tested. The regression model was fitted with a three-way interaction between radiation quality, stress, and gravity. The model was significant (AIC = 10, R^2^ = 0.7, F (15,141) = 22.08, *p* < 0.0001). The regression coefficients for each significant predictor can be found in Supplementary Table S3. Table 5 shows the analysis of variance table of the regression model for PDGF-α.

**Figure 4:**
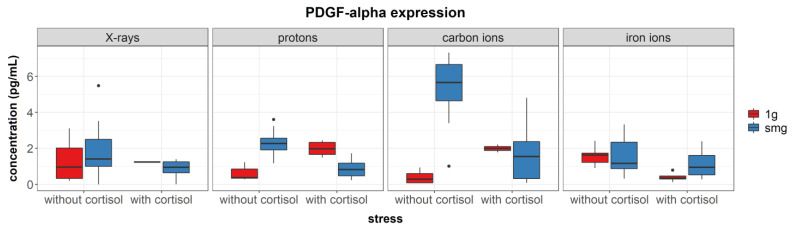
Overview of PDGF-α synthesis measured in cell lysates after exposure of NHDF to simulated microgravity, cortisol (1 µmol/L) or a combination of both as explained in Section 2.2. Measured using multiplex immunoassay. Expression values were normalized to total protein content. smg = simulated microgravity. Plot shows boxplot with median as center line, box limits are upper and lower quartiles, whisker are 1.5× interquartile range, and points are group outliers. Six replicates per condition. No effect of dose or radiation quality could be detected (see main text); therefore, data of different doses is grouped and only the effect of simulated microgravity and cortisol exposure is shown.

After exposure to simulated microgravity, a significant upregulation of PDGF-α was observed (*p* < 0.0001, Figure 4) during carbon ion experiments. However, cortisol exposure negatively interacted with simulated microgravity and reduced expression levels of PDGF-α were found in fibroblasts exposed to both simulated microgravity and cortisol, compared to simulated microgravity without cortisol exposure.

#### 3.1.4. TGF-β

During wound healing, the differentiation of fibroblasts into the more contractile myofibroblasts is stimulated through the expression of TGF-β [59]. Furthermore, its role during wound healing is of importance as it stimulates expression of ECM proteins, and migration through integrin expression [60].

Expression levels of TGF-β were examined after fibroblast had been exposed to the simulated spaceflight environment. A total of 35 outliers (relative expression > 2) were identified and removed from the data. The regression model was fitted with two three-way interactions between gravity, stress, and radiation quality, and between gravity, stress, and dose. The model was significant (AIC = −684, R^2^ = 0.29, F (27, 313) = 4.73, *p* < 0.0001). The regression coefficient for each significant predictor can be found in Supplementary Table S4. Table 6 shows the analysis of variance table of the regression model for TGF-β.

**Figure 5:**
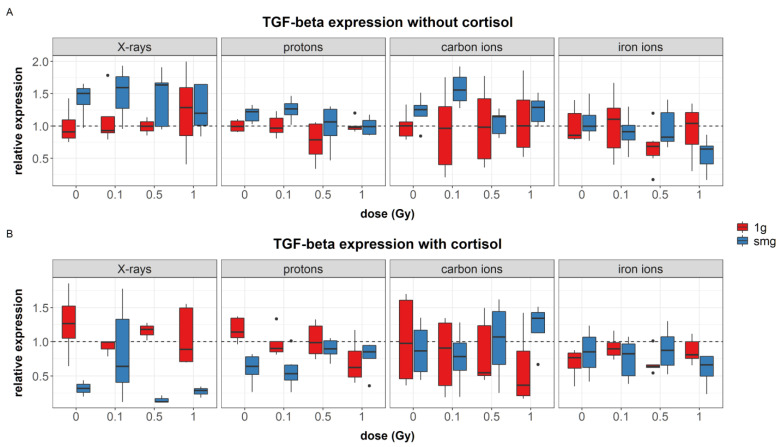
Overview of TGF-β synthesis measured in cell lysates after exposure of NHDF to simulated spaceflight stressors as explained in Section 2.2. (**A**): Effect of simulated microgravity, dose, and radiation quality on expression of TGF-β; (**B**): effect of simulated microgravity, dose, and radiation quality on expression of TGF-β in cells exposed to cortisol (1 µmol/L). Measured using ELISA immunoassay. Expression values were normalized tot total protein content, and divided by the average of 0 Gy, 1*g*, controls to obtain relative expression values. Normalized values of 0 Gy, 1*g* controls during X-rays = 1037 pg/µg, protons = 1802 pg/µg, carbon ions = 1669 pg/µg, iron ions = 2850 pg/µg. smg = simulated microgravity, dotted line = average expression of 0 Gy, 1*g*, without cortisol. Plot shows boxplot with median as center line, box limits are upper and lower quartiles, whisker are 1.5× interquartile range, and points are group outliers. Six replicates per condition.

Increased expression of TGF-β was measured after exposure to simulated microgravity (*p* = 0.0209, Figure 5A). Although, this effect was not always found (for instance during experiments with iron ions). Moreover, cortisol interacted with simulated microgravity on TGF-β expression and no upregulation was observed when cells were exposed to a combination of cortisol and simulated microgravity. This interaction effect was best observed during X-ray experiments, as the expression of TGF-β after a combined exposure to simulated microgravity and cortisol during experiments with protons, carbon ions, and iron ions was higher as compared to X-ray experiments (Figure 5B). Furthermore, at 1 Gy exposure the interaction effect between cortisol and simulated microgravity was also affected. Exposure to 1 Gy of ionizing radiation showed a trend towards lowered expression of TGF-β in cells exposed to cortisol or simulated microgravity, compared to their non-irradiated controls (*p* = 0.064 for stress * 1Gy, and *p* = 0.069 for gravity * 1Gy).

### 3.2. Effect of Simulated Spaceflight Environment on Fibroblast Migration

During the proliferation phase of wound healing, fibroblasts migrate to the site of the wound and start proliferating. Migration occurs through the extension of the actin cytoskeleton at the leading edge of the cell. Actin stress fibers, which are anchored to the ECM through focal adhesion complexes and integrins, provide the mechanical force and cell polarization needed for directional migration and contraction [8,9]. In the next section, experiments are described in which the effect of exposure to the simulated spaceflight environment on fibroblast migration and cytoskeletal rearrangements was investigated.

#### 3.2.1. Migration

**Figure 6:**
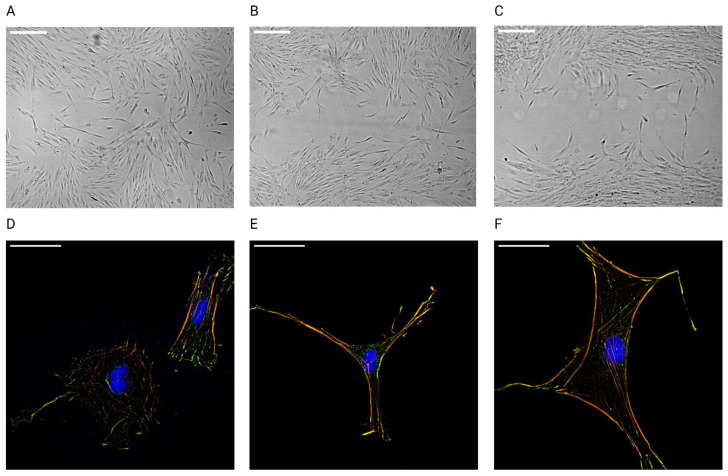
(**A**–**C**): Example images of in vitro scratch assay showing difference in wound closure after 24 h between the different conditions during iron ion experiments. Scale bar = 500µm. (**A**): 1*g*, 0 Gy, without cortisol; (**B**): 1*g*, 1 Gy, with cortisol; (**C**): simulated microgravity, 1 Gy, with cortisol; (**D**–**F**): example of different cell shapes and sizes observed at the wound edge. Immunofluorescence staining of phalloidin for actin (orange), vinculin for focal adhesions (green), and DAPI for cell nucleus (blue). Scale bar = 50 µm.

Fibroblast migration was measured through means of the in vitro scratch wound assay as described in Section 2.5. Example images of wound closure after 24 h between different conditions are shown in Figure 6A–C. Four outliers (relative closure > 2) were identified and removed from the data. The linear model was fitted with four two-way interactions between radiation quality and stress, radiation quality and dose, radiation quality and gravity, and gravity and stress. The model was significant (AIC = −3368, R^2^ = 0.25, F (24, 1102) = 15.15, *p* < 0.0001). The regression coefficients for each significant predictor can be found in Supplementary Table S5. Table 7 shows the analysis of variance table of the regression model of fibroblast migration. 

**Figure 7:**
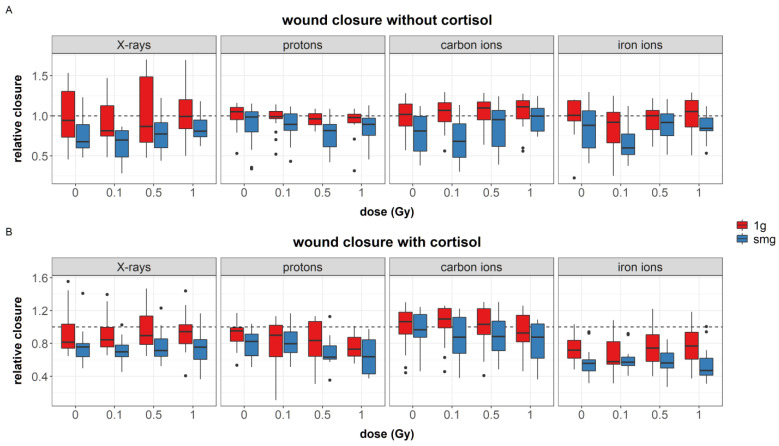
Overview of wound closure after exposure of NHDF to simulated spaceflight stressors as explained in Section 2.2. (**A**): Effect of simulated microgravity, dose, and radiation quality on cell migration; (**B**): Effect of simulated microgravity, dose, and radiation quality on cell migration after cortisol exposure (1 µmol/L). Relative closure was measured by Equation (1), values were divided by average values of 0 Gy, 1*g* controls to obtain relative values. Average relative closure values of 0 Gy, 1*g* controls during X-rays = 52%, protons = 82%, carbon ions = 73%, iron ions = 75%. smg = simulated microgravity, dotted line = average closure of 0 Gy, 1*g*, without cortisol. Plot shows boxplot with median as center line, box limits are upper and lower quartiles, whisker are 1.5× interquartile range, and points are group outliers. Six replicates per group, three images per replicate.

A significant main effect of simulated microgravity was found on wound closure, indicating a delay in migration in fibroblasts exposed to simulated microgravity (*p* < 0.0001, Figure 7A,B). During experiments with protons, however, this effect was weaker. Cortisol exposure significantly delayed wound closure during experiments with protons and iron ions. Finally, during experiments with protons, exposure to 1 Gy of protons reduced the migration capacity of fibroblasts as well.

#### 3.2.2. Cell Area

Fibroblasts are a heterogeneous cell type and at the wound edge differences in cell shape could be observed (Figure 6D,E). Exposure to growth-factors, such as TGF-β and PDGF-α, induces fibroblast differentiation in the smaller more contractile myofibroblasts [59,61]. Here, we investigated how cell area, based on the actin cytoskeleton, differed between the different exposure conditions.

A total of 15 outliers (relative dimension > 4) were identified and removed from the data. The general linear model was fitted with a four-way interaction between all simulated spaceflight stressors. The model was significant (AIC = −4544, R^2^ = 0.15, F (63, 2734) = 8.0, *p* < 0.0001). The regression coefficients for each significant predictor can be found in Supplementary Table S6. Table 8 shows the analysis of variance table for the regression model of cellular area.

**Figure 8:**
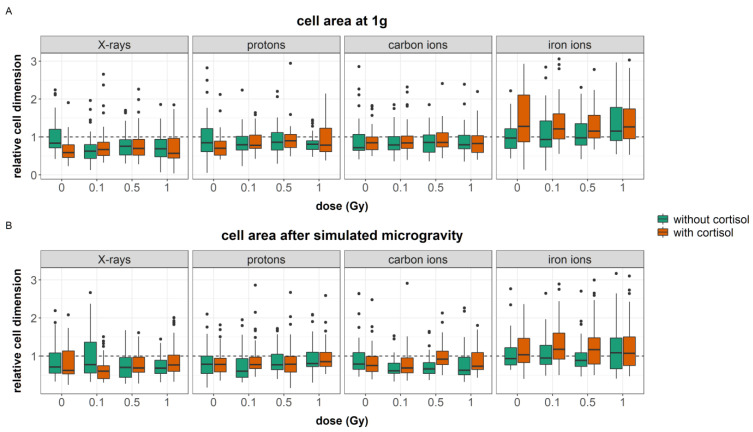
Overview of cellular surface area after exposure of NHDF to simulated spaceflight stressors as explained in Section 2.2. (**A**): Effect of cortisol (1 µmol/L), dose, and radiation quality on cellular surface area; (**B**): effect of cortisol (1 µmol/L), dose and radiation quality on cellular surface area after exposure to simulated microgravity. Dotted line represents the average closure of 0 Gy, 1*g*, without cortisol. Relative cell dimension values were obtained by dividing cell surface values by the average dimension of 0 Gy, 1*g* controls. Raw average cell surface values of 0 Gy, 1*g* controls during X-rays = 6694 µm², protons = 5057 µm², carbon ions = 5187 µm², iron ions = 4900 µm². Plot shows boxplot with median as center line, box limits are upper and lower quartiles, whisker are 1.5× interquartile range, and points are group outliers. Three replicates per condition, average of 170 cells imaged per condition.

A main effect of cortisol was found and exposure to cortisol reduced cellular surface area in fibroblasts (*p* < 0.0001). However, this cortisol-induced reduction in cell area was not observed in irradiated cells. Moreover, during experiments with carbon ions, no reducing effect of cortisol was found, and during experiments with iron ions, a significant increase in cellular surface area was observed in fibroblasts exposed to cortisol as compared to unexposed cells (Figure 8A). This indicates a batch-dependent effect of cortisol on cell area.

Reduced cellular surface area was also found in cells exposed to 0.1, 0.5, and 1 Gy of ionizing radiation. However, this effect was altered by radiation quality, and a significant increase in surface area of fibroblasts exposed to iron ions at these doses was observed. This indicates a LET-dependent dose effect on cellular area.

Exposure to simulated microgravity affected cellular area; although, the effect depended on other stressors, as for instance observed after combined exposure to simulated microgravity and cortisol, where simulated microgravity attenuated the effect of cortisol on cellular area. This effect was, however, not observed during experiments with carbon and iron ions (Figure 8B).

#### 3.2.3. Actin Stress Fibers

Actin stress fibers (Figure 6D–F) are contractile bundles of actin filaments that provide the cell with resistance for mechanical forces and are needed for cell motility and directional migration [62]. Here, we quantified the total number of actin stress fibers per cell after exposure to simulated spaceflight environment.

A total of 21 outliers (relative number > 5) were identified and removed from the data. The general linear model was fitted with a three-way interaction between dose, radiation quality, and gravity. Additionally, two two-way interactions were added. Firstly, that between stress and dose, and secondly between stress and radiation quality. Finally, as the number of actin stress fibers per cell could depend on the total cellular area and the latter was significantly affected by the different experimental conditions, total actin area per cell was added to the regression models as covariate of no-interest. The model was significant (AIC = −3750; R^2^ = 0.47, F (39, 2728) = 64.5, *p* < 0.0001, predictive R^2^ = 0.45). The regression coefficients for each significant predictor can be found in Supplementary Table S7. Table 9 shows the analysis of variance table for the regression model of number of actin stress fibers per cell.

**Figure 9:**
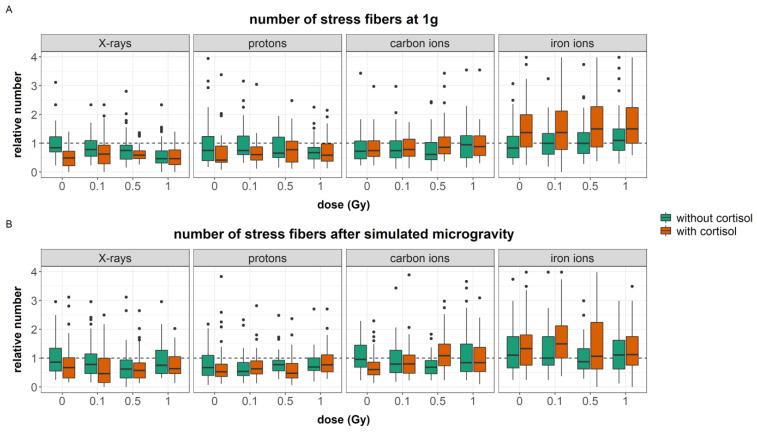
Overview of number of stress fibers per cell after exposure of NHDF to simulated spaceflight stressors as explained in Section 2.2. (**A**): Effect of cortisol (1 µmol/L), dose, and radiation quality on number of stress fibers; (**B**): effect of cortisol (1 µmol/L), dose, and radiation quality on the number of stress fibers after exposure to simulated microgravity. Dotted line = average closure of 0 Gy, 1*g*, without cortisol. Relative number values were obtained by dividing number of stress fibers in each condition by the average of 0 Gy, 1*g* controls. Raw average number of stress fibers values of 0 Gy, 1*g* controls during X-rays = 6, protons = 4, carbon ions = 4, iron ions = 4. Plot shows boxplot with median as center line, box limits are upper and lower quartiles, whisker are 1.5× interquartile range, and points are group outliers. Three replicates per condition, average of 170 cells imaged per condition.

Cortisol exposure reduced the number of stress fibers compared to unexposed cells (*p* = 0.0004, Figure 9A). However, during experiments with iron ions, the number of stress fibers per cells in fibroblasts that had been exposed to cortisol was significantly higher than during other experiments.

Exposure to 1 Gy of ionizing radiation reduced the number of stress fibers compared to non-irradiated cells. However, at higher LET, thus during carbon and iron ion exposure, this effect was opposite (Figure 9A). Additionally, simulated microgravity attenuated this response and a reduced number of stress fibers per cell was found in fibroblasts exposed to 1 Gy of iron ions and simulated microgravity (Figure 9B). Furthermore, at doses of 0.5 Gy and 1 Gy, the reducing effect of cortisol on the number of stress fibers per cell were not observed (although the effect of 1 Gy was bordering significance (*p* = 0.0927)).

Simulated microgravity alone did not significantly differ the number of stress fibers per cell. However, when combined with relatively low doses of radiation (0.1 Gy) the number of stress fibers per cell was significantly reduced compared to 1*g* controls at 0.1 Gy (Figure 9B). Again, this effect was not observed during all experiments.

#### 3.2.4. Focal Adhesions

Stress fibers are connected to the ECM through focal adhesion complexes consisting of transmembrane integrins and a set of proteins that connect the stress fibers with the integrins (Figure 6D–F). Vinculin is one of such linking protein and its key function is regulation of the force transmission within focal adhesions [63]. Per image, the total number of vinculin spots per cell were calculated.

Four outliers (relative number > 5) were identified and removed from the data. The general linear model was fitted with two three-way interactions between gravity, radiation quality, and dose, and stress, radiation quality, and dose. Furthermore, a two-way interaction between gravity and stress was added to the model as well. As can be concluded from the previous section, cell area was strongly affected by the different exposure conditions. As the number of vinculin spots per cell depends on the cellular area, the total area per cell was included in the regression model as a confounding variable. The model was significant (AIC = −6207, R^2^ = 0.67, F (64, 2725) = 114.6, *p* < 0.0001). The regression coefficients for each significant predictor can be found in Supplementary Table S8. Table 10 shows the analysis of variance table for the regression model of number of vinculin spots.

**Figure 10:**
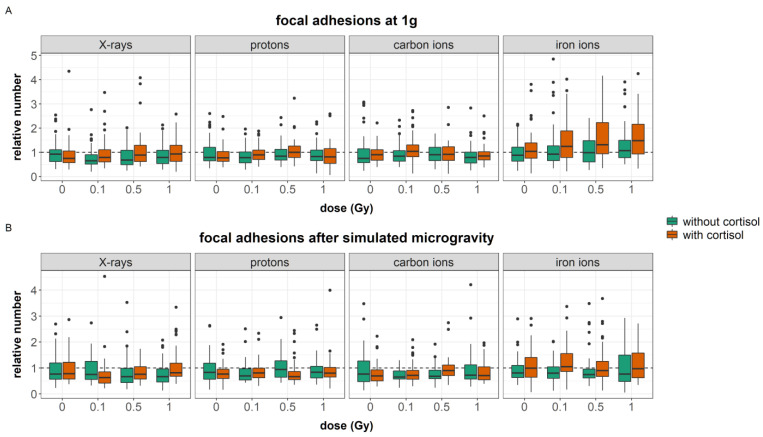
Overview of number of focal adhesions per cell after exposure of NHDF to simulated spaceflight stressors as explained in Section 2.2. (**A**): Effect of cortisol (1µmol/L), dose, and radiation quality on number vinculin spots per cell; (**B**): effect of cortisol (1µmol/L), dose, and radiation quality on number of vinculin spots per cell after exposure to simulated microgravity. Dotted line = average closure of 0 Gy, 1*g*, without cortisol. Relative number values were obtained by dividing the number of vinculin spots by average values of 0 Gy, 1*g* controls. Raw average number of vinculin spots values of 0 Gy, 1*g* controls during X-rays = 52, protons = 64, carbon ions = 62, iron ions = 61. Plot shows boxplot with median as center line, box limits are upper and lower quartiles, whisker are 1.5× interquartile range, and points are group outliers. Three replicates per condition, average of 170 cells imaged per condition.

A main effect of cortisol was observed; after cortisol exposure an increase in vinculin spots were counted (Figure 10A). However, cortisol interacted with simulated microgravity and after exposure to simulated microgravity, a reduced number of focal adhesions were observed (Figure 10B). Furthermore, cells exposed to cortisol and iron ions showed an increased number of vinculin spots with doses of 0.1, 0.5, and 1 Gy. This indicates a LET-dependent dose effect only in fibroblasts exposed to cortisol. In fibroblasts exposed to cortisol and simulated microgravity, this dose effect was, however, not observed (this four-way interaction was approaching significance (*p* = 0.06)).

During experiments where fibroblasts were exposed to protons, carbon, and iron ions, the number of vinculin spots per cell were generally higher. Furthermore, fibroblasts exposed to 1 Gy of X-rays showed an increase in the number of vinculin spots. No increase was observed during experiments with protons, carbon, and iron ions. Again, simulated microgravity reduced this effect of ionizing radiation as well.

### 3.3. Effect of Simulated Spaceflight Environment on the Synthesis of Extracellular Matrix Proteins

During the wound healing process, fibroblasts remodel the dermal matrix through expression of ECM proteins and matrix metalloproteinases. This phase can last up to several months after injury [9]. In the following section, the effect of simulated spaceflight environment on the expression of ECM proteins of fibronectin and collagen type I is investigated.

#### 3.3.1. Fibronectin

The fibronectin matrix, which is secreted by fibroblasts and endothelial cells early on during the wound healing process, serves as a scaffold for fibroblasts as they adhere to the fibronectin fibers [64]. Expression of fibronectin after exposure to the simulated spaceflight environment was measured through means of Western blot assay.

Six outliers (relative expression > 4) were identified and removed from the data. The general linear model was fitted with a three-way interaction between gravity, stress, and dose. Additionally, two two-way interactions between radiation quality and stress, and radiation quality and gravity were added to the model. The model was significant (AIC = −480, R^2^ = 0.49, F (24, 332) = 13.36, *p* < 0.0001). The regression coefficients for each significant predictor can be found in Supplementary Table S9. Table 11 shows the analysis of variance table for the regression model of fibronectin synthesis.

**Figure 11:**
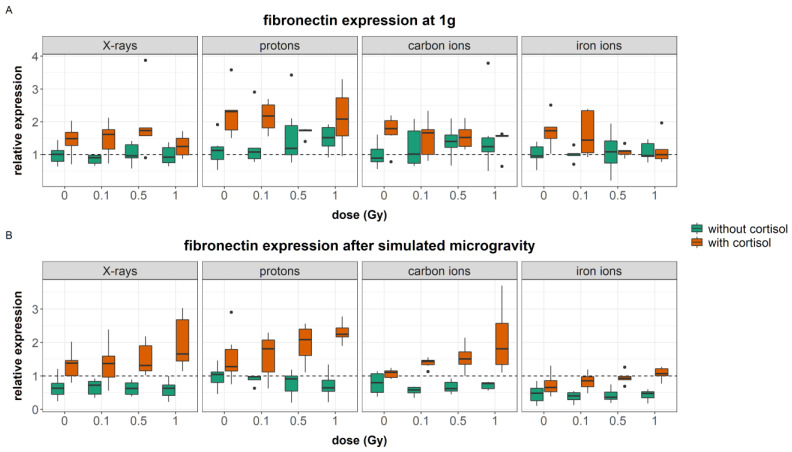
Overview of fibronectin synthesis measured in cell lysates after exposure of NHDF to simulated spaceflight stressors as explained in Section 2.2. (**A**): Effect of cortisol (1µmol/L), dose, and radiation quality on fibronectin expression; (**B**): effect of cortisol (1 µmol/L), dose, and radiation quality on fibronectin expression after simulated microgravity exposure. Measured by means of Western blot. Dotted line = average closure of 0 Gy, 1*g*, without cortisol. Raw values were normalized to total protein content, and divided by the average of 0 Gy, 1*g*, control to obtain relative expression values. Plot shows boxplot with median as center line, box limits are upper and lower quartiles, whisker are 1.5× interquartile range, and points are group outliers. Six replicates per group.

A significant main effect of cortisol exposure was found on fibroblasts expression of fibronectin. Cortisol exposure upregulated the expression of fibronectin (*p* < 0.0001, Figure 11). Fibroblasts exposed to simulated microgravity showed downregulation of fibronectin expression. During iron ion experiments, simulated microgravity downregulated the expression of fibronectin significantly (Figure 11B).

Cells that were exposed to 0.5 and 1 Gy doses of ionizing radiation in combination with cortisol showed a reduced expression of fibronectin compared to non-irradiated cells exposed to cortisol (Figure 11A). However, when cortisol exposure was combined with simulated microgravity exposure, an increase in fibronectin expression was observed at 0.5 and 1 Gy (Figure 11B). This indicates that ionizing radiation can influence the expression of fibronectin in fibroblasts when cells are also exposed to cortisol. Yet, the effect strongly depended on the gravity level.

#### 3.3.2. Collagen Type I

During the remodeling phase of wound healing, type III collagen is broken down and replaced by type I collagen, which provides the wound with more tensile strength [9]. Here, we measured the expression of procollagen type I alpha 1 (α1) and alpha 2 (α2) after exposure to simulated spaceflight environment through means of Western blot assay.

For procollagen type I α1, 14 outliers (relative expression > 4) were identified and removed from the data. The regression model was fitted with three two-way interactions between stress and radiation quality, stress and gravity, and gravity and radiation quality. The model was significant (AIC = −248, R^2^ = 0.45, F (12, 257) = 17.79, *p* < 0.0001). The regression coefficients for each significant predictor can be found in Supplementary Table S10. Table 12 shows the analysis of variance table for the regression model of type I α1 procollagen synthesis.

**Figure 12:**
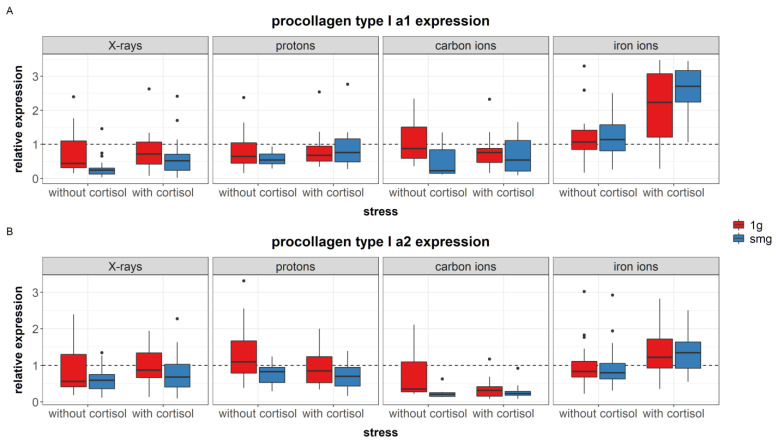
Overview of procollagen type I α1 (**A**) and α2 (**B**) synthesis measured in cell lysates after exposure of NHDF to simulated microgravity and cortisol (1 µmol/L) as explained in Section 2.2. smg = simulated microgravity, dotted line = average expression of 0 Gy, 1*g*, without cortisol. Raw values were normalized to total protein content, and divided by the average of 0 Gy, 1*g*, control to obtain relative expression values. Plot shows boxplot with median as center line, box limits are upper and lower quartiles, whisker are 1.5× interquartile range, and points are group outliers. Dose did not significantly affect the synthesis of both procollagen type I α1 and α2. Therefore, data are grouped for the different doses. Six replicates per condition.

A significant main effect of simulated microgravity was found, which lowered the expression of procollagen type I α1 (*p* = 0.0277, Figure 12A). During experiments with iron ions, this effect of simulated microgravity was, however, not observed. Furthermore, exposure to cortisol during iron ion experiments increased the expression of procollagen type I α1.

For procollagen type I α2, four outliers (relative expression > 4) were identified and removed from the data. The model was fitted with a two-way interaction between stress and radiation quality, and a main effect of gravity. The model was significant (AIC = −367, R^2^ = 0.25, F (8, 278) = 11.38, *p* < 0.0001). The regression coefficients for each significant predictor can be found in Supplementary Table S11. Table 13 shows the analysis of variance table for the regression model of type I α2 procollagen synthesis.

Significant differences between experiments were found in the expression levels of procollagen type I α2. In general, expression levels during proton and iron ion experiments were higher compared to experiments with X-rays and carbon ions.

A significant main effect of simulated microgravity was found, which lowered the expression of procollagen type I α2 (*p* = 0.0007, Figure 12B). During experiments with protons, cortisol exposure significantly reduced the expression of procollagen type I α2 as well.

## 4. Discussion

In this paper, an *in vitro* simulation of the spaceflight environment has been used to investigate the effect on the fibroblasts’ wound healing capacity. The effect of simulated microgravity, ionizing radiation of several radiation qualities and at different doses, and stress hormones was investigated for each spaceflight stressor individually and in combination. Different functions of fibroblasts related to the wound healing process have been studied, covering the inflammation, proliferation, and remodeling phases. In the following sections, we will discuss how the different spaceflight stressors may interact and act upon the wound healing process.

### 4.1. Interaction of Simulated Microgravity and Cortisol during the Inflammatory Phase of Wound Healing

#### 4.1.1. Simulated Microgravity Effects

Simulated microgravity upregulated the expression of the growth factors PDGF-α (during experiments with carbon ions) and TGF-β. Furthermore, upregulation of IL-6 was seen during carbon ion experiments and IL-1RA seemed to be upregulated after exposure to simulated microgravity as well; although this main effect was not significant. Increased levels of TGF-β1 gene expression and IL-6 concentrations have been identified in endothelial cells after exposure to simulated microgravity; although no measurable effect was observed in TGF-β1 concentrations [65]. Furthermore, increased excretion of IL-6 has been found in astronauts’ urine samples during the first day of spaceflight [66].

PDGF-α works as a chemoattractant and enhances the proliferation of fibroblasts during later phases of wound healing. Furthermore, it stimulates the expression of ECM proteins and contraction of the wound [61]. As PDGF-α and TGF-β show a synergistic relationship [67], it is not surprising to also find increased levels of TGF-β in fibroblasts exposed to simulated microgravity. Although TGF-β1 during wound healing is important for fibroblast differentiation and stimulation of ECM protein expression, its inflammatory effect in the skin has been related to delayed wound healing in transgenic mice [68].

IL-6 also plays an important role during the wound healing process. It works as a mitogen compound and IL-6-deficient mice show impaired wound healing [58]. High levels of IL-6 can also be found in dermal and epidermal cells of psoriatic plaques [69]. Injection of IL-6 results in erythema and an infiltration of lymphocytes in the dermis, which is indicative of inflammatory changes as the result of high levels of IL-6 [70]. Additionally, IL-1RA regulates the wound healing process at several phases. Its absence in knockout mice is linked to delayed healing, as shown by delayed granulation tissue formation and neovascularization. Furthermore, reduced levels of type I collagen gene expression were also indicated in these mice [57]. As IL-1RA competitively binds to the IL-1 receptor, the balance between IL-1RA and IL-1 is important for skin health. An increased IL-1RA/IL-1 ratio has been found in sun-exposed skin as well as in biopsies taken from skin sites of patients with inflammatory cutaneous disorders [71].

Although all aforementioned cytokines and growth-factors are crucial for proper wound healing, overexpression can be linked to inflammatory skin conditions [69,70,71]. Moreover, despite their stimulating function for fibroblast migration, a significantly delayed migration of fibroblasts exposed to simulated microgravity was observed in this study. This suggests that the increased expression of cytokines and growth factors that were observed in simulated microgravity either dampens fibroblast migration, or alternatively, the upregulation of these cytokines and growth factors is not sufficient to promote cell migration under simulated microgravity conditions.

#### 4.1.2. Combined Effects

Besides the increase in cytokine and growth factor expression in fibroblasts exposed to simulated microgravity, cortisol decreased the expression of IL-6 and IL-1RA. Moreover, when cells were exposed to simulated microgravity in combination with cortisol, the upregulation of cytokine and growth factors, as observed in simulated microgravity only, was not found. Likewise, in some conditions, exposure to ionizing radiation also lowered the expression levels in cells exposed to simulated microgravity, as for instance seen after 1 Gy of iron ion exposure where IL-1RA expression in fibroblasts exposed to simulated microgravity was significantly lower as compared to the other groups in simulated microgravity. On the contrary, after exposure to 1 Gy of iron ions in fibroblasts that have also been exposed to a combination of simulated microgravity and cortisol, a significant increase in IL-6 expression was observed compared to the other groups. This indicates that, although simulated microgravity may upregulate the expression of certain cytokines and growth factors, when the effect of other spaceflight stressors is taken into account, the expression levels may alter. Furthermore, the effect of ionizing radiation may differ depending on whether fibroblasts have been exposed to a set of spaceflight stressors or one spaceflight stressor in isolation, and ultimately, this effect can furthermore depend on the radiation quality (LET and ion).

### 4.2. Fibroblast Migration and Cytoskeletal Remodeling Is Affected by Simulated Spaceflight Stressors

The effect of simulated microgravity on cell migration can be complex and even contradictory [27]. In this paper, we have shown with a large dataset that simulated microgravity reduced the migration capacity of human dermal fibroblasts regardless of dose and cortisol exposure and for all experiments (Figure 7). Simulated microgravity alone did not significantly alter cellular area or the number of focal adhesions or stress fibers. This is in contradiction with the loss of stress fibers observed in mouse osteoblasts and human epidermoid cancer cells during actual spaceflight [72,73] and in human umbilical vein endothelial cells and human mesenchymal stem cells during simulated microgravity conditions using the RPM or rotating wall vessel system [74,75,76,77,78,79]. Likewise, reduced motility of J-111 monocytes during spaceflight was linked to a disruption of actin fibers [80]. Nevertheless, in our study, simulated microgravity interacted with cortisol and ionizing radiation at several endpoints. Cortisol exposure increased the number of focal adhesions. Furthermore, an increased cellular area was found in fibroblasts exposed to cortisol as compared to unexposed controls during carbon and iron ion experiments. Both cortisol-induced effects were not observed after exposure to a combination of cortisol and simulated microgravity. Furthermore, the effects of ionizing radiation on both the number of stress fibers and focal adhesions per cell were attenuated by simulated microgravity as well. This indicates that, although observations may show no main effect of simulated microgravity on cytoskeletal remodeling, the spaceflight stressor can interact with and influence the effect of other spaceflight stressors.

Similar to simulated microgravity, cortisol also reduced the migration capacity of fibroblasts, yet this effect was not always significant. This might be due to the dynamic aspect of fibroblast migration and the experimental design that was used in this study, where the observation of wound closure is only done at one specific time point. This notion is further supported by the data shown in Radstake et al. [81], where measuring the migration of fibroblasts over a longer period of time shows an initial delay in migration, while at later time points, migration capacity and wound closure is recovered.

The cortisol-induced delayed migration was best observed during experiments with iron ions. During these experiments, fibroblasts had a larger cellular area, an increased number of stress fibers, and a reduced number of focal adhesions. The latter endpoint significantly increased with dose as a result of exposure to both cortisol and iron ions. Actin stress fibers are crucial for the contractility of cells needed for motility and proper migration [62]. Cortisol has the ability to interact with and remodel the actin cytoskeleton, resulting in increased thickness and stability after exposure to glucocorticoids [82]. Alterations in the number of actin stress fibers are linked to cellular stiffness, and a loss of actin stress fibers is linked to reduced cellular stiffness and migration capacity [83]. Focal adhesions link actin stress fibers to the surrounding ECM. During migration, a remodeling of stress fibers and disassembly of focal adhesions is needed for the retraction of the rear part in migrating cells [62]. However, increased cytoskeletal stiffening, as observed with increased number of stress fibers, leads to a strengthening of focal adhesions [84].

In the results presented in this paper, the increased number of actin stress fibers and vinculin spots as a result of exposure to cortisol either with or without exposure to ionizing radiation as observed during iron ion experiments was linked to a reduced migration. This suggests that the increased number of stress fibers resulting from cortisol exposure, together with increased focal adhesion after exposure to cortisol in combination with iron ions as well as the increased area of the cells, are linked to reduced migratory capacity possibly due to the increased adhesion of cells to the substrate. This supports the notion of a “sweet-spot” of the number of actin fibers where dynamic remodeling of the actin cytoskeleton supports cellular motility. However, an increase in the number of fibers may fix cells to the substratum and limit their motility, especially when cellular dimensions are larger and an increased number of focal adhesions is present as well. The difference between the experiments suggests that the effect of cortisol on migration and number of actin stress fibers is not chronic, and timing differences may exist, as also observed in migration behavior.

Additionally, in this paper, a reduced expression of IL-6 was observed after cortisol exposure. IL-6 has been indicated to stimulate migration in smooth muscle cells by inducing cytoskeletal reorganization and induction of F-actin stress fibers [85]. The reduced migration found after cortisol exposure, together with lower levels of IL-6, observed cytoskeletal differences between cortisol exposed cells, and unexposed controls further supports the notion of the effect of IL-6 on cytoskeletal rearrangements needed for migration.

Finally, exposure to 1 Gy of protons led to a reduced migration and lower expression of vinculin spots in fibroblasts regardless of exposure to other simulated spaceflight stressors. Ionizing radiation has been shown to be able to affect the migration capacity of cells, and both an increase and decrease can be observed after exposure to ionizing radiation. Moreover, ionizing radiation effects on cell migration are dependent on cell type, total dose, as well as timing (as reviewed in Verde et al. [86]). In transformed fibroblasts, 1 Gy of X-ray exposure inhibited cell migration, which was explained by radiation-induced changes in cytoskeletal structure. However, in healthy cell lines, this effect was only observed at higher doses [87]. Based on the results in this paper, ionizing radiation exposure leads to changes in the number of actin filaments and vinculin spots; although the effect depends on the radiation quality. Moreover, an effect of radiation quality on the cell dimension could be observed as well, where 1 Gy exposure to photons led to smaller cell bodies. Interestingly, exposure to 1 Gy of iron ions led to an increase in cellular dimension. However, this did not lead to observable changes in migration capacity for all groups. This suggests that, although ionizing radiation can induce changes in cytoskeletal architecture, which could lead to observable changes in cell migration, at doses relevant for space, the induced changes are reversible and not strong enough to affect the migration of fibroblasts, which was more strongly affected by other spaceflight stressors.

### 4.3. Simulated Spaceflight Stressors Interact and Affect the Expression of Dermal Matrix Proteins in Fibroblasts

#### 4.3.1. Simulated Microgravity Effects

Simulated microgravity downregulated the expression of fibronectin and procollagen type I in fibroblasts. This is in accordance with the literature where exposure to simulated microgravity reduced gene expression levels of fibronectin in fibroblasts after RPM exposure [88], as well as type I collagen protein expression in osteoblasts (rotating wall vessel) [89] and fibroblasts after RPM exposure [48]. However, an upregulation of fibronectin can also be found in fibroblasts after simulated microgravity exposure using the RPM [48]. On the contrary, during experiments with iron ions, an upregulation of procollagen type I α1 was found after simulated microgravity exposure. The 3D co-cultures of fibroblasts and keratinocytes have indicated an upregulation of type I procollagen α1 after exposure to simulated microgravity for 7 days with the use of a 3D clinostat [90]. Furthermore, transcriptomic analyses of skin tissue from space-flown mice show increased expression levels of procollagen compared to ground controls [91], and increased collagen content has also been found in astronauts after spaceflight [30]. This indicates that type I procollagen is sensitive to simulated microgravity; although, its effect can differ depending on the complexity of the *in vitro* and *in vivo* model. Furthermore, contradictory results may be explained by slight differences in timing of exposures or sample collection after simulated microgravity exposure. More data at different time points would be needed to better understand the time-dependent response of fibroblasts procollagen type I expression under simulated microgravity. Furthermore, although expression levels of procollagens can be affected, it cannot be concluded from the results presented in this study how this affects collagen fiber formation and deposition in the ECM. Moreover, although excretion of procollagens can be increased as result of spaceflight, ECM degradation can still be observed, possibly due to excessive degradation of the newly formed procollagen [91].

#### 4.3.2. Combined Effects

In fibroblasts exposed to cortisol, fibronectin expression was significantly higher compared to controls. This finding is in agreement with the literature, as other studies have also found that treatment of fibroblasts cultures with cortisol induced upregulation of fibronectin [92,93,94]. Moreover, when fibroblasts were exposed to simulated microgravity in combination with cortisol, a dose-dependent effect could be observed with a significant increase in fibronectin expression at higher doses of ionizing radiation. Besides the upregulation of fibronectin, in the case of iron ion experiments, procollagen type I was also upregulated in fibroblasts exposed to simulated microgravity and/or cortisol (although no dose-dependent effects could be established). High levels of fibronectin as well as collagen type I are linked to the formation of keloids and hypertrophic scars and are an indication of abnormal wound healing [92,95]. Expression of growth-factors TGF-β1 and PDGF-α induce the expression of fibronectin during wound healing [60,61,96]. However, the dose-dependent upregulation of fibronectin in fibroblasts exposed to simulated microgravity and cortisol in these experiments was not linked to an increased expression of these growth factors and could, therefore, not be explained based on current data. More research is, therefore, needed to better understand these observations. Nevertheless, observation of the dose-dependent increase in fibronectin expression, which was most clearly observed in fibroblasts exposed to both simulated microgravity and cortisol, suggests that the complete simulated spaceflight environment could influence matrix deposition during wound healing, mostly when all three spaceflight stressors are taken into account. In turn, this could lead to increased matrix deposition and excessive scar formation as a result of exposure to the spaceflight environment.

### 4.4. Limitations and Future Directions

In this study, we have developed and tested an *in vitro* model of the spaceflight environment. After exposure of dermal fibroblasts to this simulated spaceflight environment, we observed findings such as altered expression of cytokines, cytoskeletal remodeling, and ECM protein expression, which are in line with findings from spaceflight studies. While the results in this paper help to improve our understanding on the interaction of different spaceflight stressors on the fibroblasts function during wound healing and provide a starting point for future experiments, some limitations remain, which should be addressed.

The chosen endpoints in this study represent some of the important functions of fibroblasts relevant for proper wound healing and skin integrity. However, the wound healing process consists of many components, and therefore, other parameters, which have not been considered in this study, can be studied in future experiments. The presented *in vitro* model can be used to study other skin parameters, such as collagen type VII, a less abundant protein found in the skin, which, nevertheless, plays a crucial role in the skin’s integrity and wound healing [97]. In addition, cytoskeletal structures of microtubuli and vimentin have previously been found to be altered during spaceflight [98,99,100]. Other factors involved in the remodeling of the skin, such as matrix metalloproteinases, can also be considered [101]. Therefore, it would be of interest to apply the described model of the simulated spaceflight environment to investigate other endpoints as well.

While the diverse role of skin fibroblasts during the wound healing process makes them a suitable *in vitro* model for studying the effects of simulated spaceflight stressors on the wound healing process, this monoculture does not account for the complexity of the skin and the interaction between fibroblasts and other skin cells. The skin is a neuroendocrine system, which means that a complex interaction exists between the nervous system, endocrine system, and immune system. This complexity serves to maintain proper barrier function of the skin [102]. As a result, fibroblasts’ function is regulated by a plethora of different cell types. For example, dysregulation of the immune system in microgravity might contribute to the increases in skin sensitivity as observed during spaceflight [103,104,105]. Furthermore, generalization of the present results remains limited as fibroblasts were obtained from one donor. For these reasons, the present study does not account for inter-individual differences or skin complexity as found *in vivo*. To explore these effects, the experiments could be repeated with cells obtained from multiple donors, and in more complex systems, such as organotypic skin cultures, skin-on-a-chip, and *in vivo* models.

Another limitation of the applied methodology in this study is the use of the RPM to simulate the microgravity environment. While this method is a well-accepted method to study biological processes in cells that are dependent on the gravity vector, it comes, however, with constraints. Gravity artifacts may arise due to RPMs kinematic rotation, and hence, a moderate velocity and distance from the center of rotation should be considered [106]. In addition, fluid motion inside the flasks due to the accelerations causes shear stress on cells attached to the vessel wall. Studies have shown that the amount of mechanical forces due to shear stress are higher on the RPM compared to clinostats, which differently affected cellular responses to the simulated microgravity environment [107,108]. While moderate velocity settings were chosen in this study, some effects of shear forces would remain and should be taken into account when interpreting these results. High shear stress influences fibroblasts arrangements and function related to wound healing and high shear stress has been related with increased migration speed in fibroblasts [109]. Therefore, in future studies, to validate these results, the study should be repeated using real microgravity platforms.

With an eye on future interplanetary space missions, it is of great importance to develop effective countermeasures and protect astronauts against possible infections resulting from delayed wound healing. The development of wound dressings that promote healing and reduce risks of infections, but at the same time have a long shelf-life and take up little space, could hold promises to this aim. Of interest is the finding that treatment of wounds with platelet-rich plasma (PRP) in a wound healing model of the leech was shown to be successful in counteracting the delayed healing as result of simulated microgravity exposure [46]. This model may be used to further test the effectiveness of PRP as a treatment to restore wound healing in simulated microgravity combined with radiation and cortisol exposure as well. In addition, it would be of interest to investigate if and how the skin-related spaceflight effects return back to normal once astronauts are back on Earth, or at partial gravity of the Moon and Mars. For these aims, the current *in vitro* model could provide a methodology to study the effect of exposure to changing gravity fields on the *in vitro* wound healing process. Finally, with the increase in both commercial and touristic spaceflight, efforts should be made to reduce the risks of obtaining skin injuries as a result of, for instance, friction induced by spacesuits.

## 5. Conclusions

To the best of our knowledge, this study is the first to combine simulated microgravity, ionizing radiation, and cortisol to simulate the spaceflight environment *in vitro*. Using this model, we have investigated how wound healing capacities of dermal fibroblasts were affected after exposure to this set of spaceflight stressors. It can be concluded from our study that the simulated spaceflight environment can affect fibroblast wound healing capacity at any phase during the wound healing process. Moreover, as shown by the interaction effect between simulated microgravity and cortisol, the effects of exposure to one single spaceflight stressor can be altered when other spaceflight stressors are considered as well. Finally, some spaceflight stressors, such as ionizing radiation, only showed effects when fibroblasts were exposed to simulated microgravity and cortisol as well, while the response could depend on the radiation quality.

The interaction of the different spaceflight stressors highlights the complexity that needs to be taken into account when studying the effect of spaceflight on certain biological processes. Furthermore, the wound healing process consists of a complex and delicate interaction between different cellular components and phases. Fibroblasts play an important role during the wound healing process and their sensitivity to exposure to simulated spaceflight stressors makes this important function of the skin especially vulnerable under spaceflight conditions. Countermeasures should be developed to reduce the risk of delayed and impaired healing, which challenges the barrier function of the skin and increases the risk of infections and health complications.

## Figures and Tables

**Table 1:** Overview of the radiation type, energy, LET, and microdosimetric quantities.

Radiation Type	Energy	LET _D_, _Primary_ [keV/µm]	LET _D, All_ [keV/µm]	y_D_ [keV/µm]
H-250 X-rays	Supplementary Materials	-	0.4	4.0
^1^ H ions	150 MeV	0.56	3.8	5.0
^12^ C ions	90 MeV/n	28.2	29.3	18.1
^56^ Fe ions	1000 MeV/n	155	155	73.1

More details can be found in the Supplementary Materials.

**Table 2:** Overview of regression model variables.

Categorical Variable	Levels	Notation
Stress		
	Control vehicle	
	Cortisol	(x_cort_)
Gravity		
	1*g*	
	Simulated microgravity	(x_smg_)
Dose		
	0 Gy	
	0.1 Gy	(x_d0.1_)
	0.5 Gy	(x_d0.5_)
	1 Gy	(x_d1_)
Radiation quality		
	Photons	
	Protons	(x_prot_)
	Carbon ions	(x_C_)
	Iron ions	(x_Fe_)

**Table 3:** ANOVA table for the regression model of IL-1RA.

Source of Variation	Df	Sum Sq	F Value	*p* Value
stress	1	3.90	97.70	<0.0001
gravity	1	2.19	54.88	<0.0001
dose	3	1.38	11.59	<0.0001
radiation quality	3	2.05	17.13	<0.0001
stress * gravity	1	0.68	17.28	<0.0001
stress * dose	3	0.01	0.11	0.9523
gravity * dose	3	0.21	1.83	0.1416
stress * radiation quality	3	0.26	2.21	0.0868
gravity * radiation quality	3	0.66	5.55	0.0010
dose * radiation quality	9	0.75	2.10	0.0288
stress * gravity * dose	3	0.03	0.27	0.8408
stress * gravity * radiation quality	3	0.03	0.27	0.8414
stress * dose * radiation quality	9	1.06	2.96	0.0021
gravity * dose * radiation quality	9	0.78	2.19	0.0222
stress * gravity * dose * radiation quality	9	0.68	1.91	0.0497
residuals	305	12.17		

Df = degree of freedom, Sum Sq = sum of squares, * indicates interaction effects between indicated variables.

**Table 4:** ANOVA table for the regression model of IL-6.

Source of Variation	Df	Sum Sq	F Value	*p* Value
stress	1	30.80	215.50	<0.0001
gravity	1	0.30	2.07	0.1516
dose	3	1.55	3.62	0.0138
radiation quality	3	7.98	18.62	<0.0001
stress * gravity	1	0.03	0.22	0.6405
stress * dose	3	0.12	0.28	0.8380
gravity * dose	3	0.79	1.84	0.1408
stress * radiation quality	3	5.54	12.91	<0.0001
gravity * radiation quality	3	4.48	10.46	<0.0001
dose * radiation quality	9	0.69	0.54	0.8478
stress * gravity * dose	3	0.71	1.65	0.1775
stress * gravity * radiation quality	3	2.99	6.98	0.0002
stress * dose * radiation quality	9	1.39	1.08	0.3747
gravity * dose * radiation quality	9	1.39	1.08	0.3790
stress * gravity * dose * radiation quality	9	4.18	3.25	0.0009
residuals	258	36.87		

Df = degree of freedom, Sum Sq = sum of squares, * indicates interaction effects between indicated variables.

**Table 5:** ANOVA table for the regression model of PDGF-α.

Source of Variation	Df	Sum Sq	F Value	*p* Value
stress	1	67.53	69.50	<0.0001
gravity	1	29.74	30.61	<0.0001
radiation quality	3	32.54	33.48	<0.0001
gravity * stress	1	34.83	35.84	<0.0001
gravity * radiation quality	3	9.31	9.58	<0.0001
stress * radiation quality	3	11.44	11.77	<0.0001
gravity * stress * radiation quality	3	9.98	10.27	<0.0001
residuals	141	0.97		

Df = degree of freedom, Sum Sq = sum of squares, * indicates interaction effects between indicated variables.

**Table 6:** ANOVA table for the regression model of TGF-β.

Source of Variation	Df	Sum Sq	F Value	*p* Value
stress	1	4.33	34.82	<0.0001
gravity	1	0.00	0.00	0.9812
dose	3	0.14	1.12	0.3401
radiation quality	3	0.74	5.92	0.0006
stress * gravity	1	2.24	18.05	<0.0001
stress * radiation quality	3	0.37	2.99	0.0314
gravity * radiation quality	3	0.50	4.05	0.0076
stress * dose	3	0.13	1.04	0.3751
gravity * dose	3	0.11	0.90	0.4435
stress * gravity * radiation quality	3	0.80	6.41	0.0003
stress * gravity * dose	3	0.31	2.49	0.0605
residuals	313	0.12		

Df = degree of freedom, Sum Sq = sum of squares, * indicates interaction effects between indicated variables.

**Table 7:** ANOVA table for the regression model of fibroblast migration.

Source of Variation	Df	Sum Sq	F Value	*p* Value
radiation quality	3	4.05	27.37	<0.0001
stress	1	2.61	53.01	<0.0001
dose	3	0.42	2.87	0.0352
gravity	1	6.96	141.33	<0.0001
radiation quality * stress	3	2.50	16.89	<0.0001
radiation quality * dose	9	0.85	1.92	0.0450
radiation quality * gravity	3	0.40	2.71	0.0440
stress * gravity	1	0.12	2.37	0.1241
residuals	1102	54.29		

Df = degree of freedom, Sum Sq = sum of squares, * indicates interaction effects between indicated variables.

**Table 8:** ANOVA table for the regression model of cell dimension.

Source of Variation	Df	Sum Sq	F Value	*p* Value
stress	1	2.15	11.11	0.0009
gravity	1	1.18	6.07	0.0138
dose	3	0.38	0.66	0.5778
radiation quality	3	68.12	117.24	<0.0001
stress * gravity	1	0.05	0.28	0.5974
stress * dose	3	2.00	3.44	0.0162
gravity * dose	3	0.29	0.50	0.6799
stress * radiation quality	3	5.94	10.22	<0.0001
gravity * radiation quality	3	1.95	3.36	0.0180
dose * radiation quality	9	3.57	2.05	0.0310
stress * gravity * dose	3	0.51	0.88	0.4483
stress * gravity * radiation quality	3	0.49	0.85	0.4683
stress * dose * radiation quality	9	3.93	2.25	0.0166
gravity * dose * radiation quality	9	1.98	1.14	0.3314
stress * gravity * dose * radiation quality	9	5.55	3.18	0.0008
residuals	2743	531.22		

Df = degree of freedom, Sum Sq = sum of squares, * indicates interaction effects between indicated variables.

**Table 9:** ANOVA table for the regression model of number of stress fibers per cell.

Source of Variation	Df	Sum Sq	F Value	*p* Value
actin area	1	424.05	1667.50	<0.0001
dose	3	0.22	0.28	0.8386
radiation quality	3	149.58	196.07	<0.0001
gravity	1	0.05	0.21	0.6451
stress	1	0.95	3.75	0.0530
dose * radiation quality	9	5.68	2.48	0.0081
dose * gravity	3	0.96	1.26	0.2857
radiation quality * gravity	3	2.08	2.73	0.0427
dose * stress	3	2.17	2.84	0.0364
radiation quality * stress	3	18.28	23.97	<0.0001
dose * radiation quality * gravity	9	6.27	2.74	0.0035
residuals	2728	693.74		

Df = degree of freedom, Sum Sq = sum of squares, * indicates interaction effects between indicated variables.

**Table 10:** ANOVA table for the regression model of number of vinculin spots.

Source of Variation	Df	Sum Sq	F Value	*p* Value
actin area	1	528.77	4983.95	<0.0001
stress	1	6.10	57.51	<0.0001
gravity	1	11.43	107.72	<0.0001
radiation quality	3	31.73	99.69	<0.0001
dose	3	1.00	3.13	0.0247
stress * gravity	1	2.39	22.53	<0.0001
gravity * radiation quality	3	1.73	5.44	0.0010
gravity * dose	3	1.11	3.48	0.0152
radiation quality * dose	9	2.55	2.67	0.0043
stress * radiation quality	3	3.60	11.30	<0.0001
stress * dose	3	0.13	0.42	0.7358
gravity * radiation quality * dose	9	2.32	2.43	0.0095
stress * radiation quality * dose	9	3.04	3.18	0.0008
residuals	2739	290.60		

Df = degree of freedom, Sum Sq = sum of squares, * indicates interaction effects between indicated variables.

**Table 11:** ANOVA table for the regression model of fibronectin synthesis.

Source of Variation	Df	Sum Sq	F Value	*p* Value
stress	1	34.32	141.01	<0.0001
gravity	1	11.76	48.30	<0.0001
dose	3	1.41	1.94	0.1237
radiation quality	3	16.28	22.29	<0.0001
stress * gravity	1	2.69	11.04	0.0010
stress * dose	3	0.30	0.40	0.7496
gravity * dose	3	1.24	1.69	0.1685
stress * radiation quality	3	2.64	3.62	0.0135
gravity * radiation quality	3	1.80	2.46	0.0624
stress * gravity * dose	3	5.62	7.70	<0.0001
residuals	332	80.82		

Df = degree of freedom, Sum Sq = sum of squares, * indicates interaction effects between indicated variables.

**Table 12:** ANOVA table for the regression model of type I α1 procollagen synthesis.

Source of Variation	Df	Sum Sq	F Value	*p* Value
stress	1	6.83	17.99	<0.0001
radiation quality	3	55.63	48.81	<0.0001
gravity	1	1.82	4.80	0.0294
stress * radiation quality	3	11.99	10.52	<0.0001
stress * gravity	1	1.96	5.16	0.0240
radiation quality * gravity	3	2.85	2.50	0.0603
residuals	257	97.64		

Df = degree of freedom, Sum Sq = sum of squares, * indicates interaction effects between indicated variables.

**Table 13:** ANOVA table for the regression model of type I α2 procollagen synthesis.

Source of Variation	Df	Sum Sq	F Value	*p* Value
stress	1	0.02	0.07	0.7893
radiation quality	3	17.71	21.93	<0.0001
gravity	1	3.30	12.24	0.0005
Stress * radiation quality	3	3.48	4.31	0.0054
residuals	278	74.86		

Df = degree of freedom, Sum Sq = sum of squares, * indicates interaction effects between indicated variables.

## Data Availability

Data available upon request.

## Supplementary Materials

The following supporting information can be downloaded at: https://www.mdpi.com/article/10.3390/cells12020246/s1 [110]. Figure SM1: Normalized energy spectrum of the H250 X-rays used for the cell exposures at SCK CEN, Mol, Belgium; Supplementary Table 1: Significant estimates for the expression of IL-1RA; Supplementary Table 2: Significant estimates for the expression of IL-6; Supplementary Table 3: Significant estimates for the expression of PDGF-α; Supplementary Table 4: Significant estimates for the expression of TGF-β; Supplementary Table 5: Coefficients of significant estimates for fibroblast migration; Supplementary Table 6: Coefficients of significant estimates for actin area after exposure to simulated spaceflight stressors; Supplementary Table 7: Coefficients of significant estimates for number of stress fibers per cell; Supplementary Table 8: Coefficients of significant estimates for the number of vinculin spots; Supplementary Table 9: Coefficients of significant estimates for the synthesis of fibronectin; Table 10: Coefficients of significant estimates for the expression of type I α1 procollagen; Supplementary Table 11: Coefficients of significant estimates for the expression of type I α2 procollagen.
