# Supplementary material for: The Effects of Combined Exposure to Simulated Microgravity, Ionizing Radiation, and Cortisol on the In Vitro Wound Healing Process
Source: Cells. 2023 Jan 7;12(2):246. doi: 10.3390/cells12020246 (PMC9857207; doi:10.3390/cells12020246)
Supplement: Supplementary file 1 [file cells-12-00246-s001.zip › cells-2072073-supplementary.pdf]

SUPPLEMENTARY MATERIALS TO

The Effects of Combined Exposure to Simulated Microgravity, Ionizing Radiation, and Cortisol on the *In Vitro* Wound Healing Process

Wilhelmina E. Radstake [1,2], Kiran Gautam [1], Silvana Miranda [1,2], Randy Vermeesen [1], Kevin Tabury [1,3], Emil Rehnberg [1,2], Jasmine Buset [1], Ann Janssen [1], Liselotte Leysen [1], Mieke Neefs [1], Mieke Verslegers [1], Jürgen Claesen [4,5], Marc-Jan van Goethem [6], Uli Weber [7], Claudia Fournier [7], Alessio Parisi [8,9], Sytze Brandenburg [6], Marco Durante [7,10], Bjorn Baselet [1,*] and Sarah Baatout [1,2]

SM1. X-ray energy spectrum

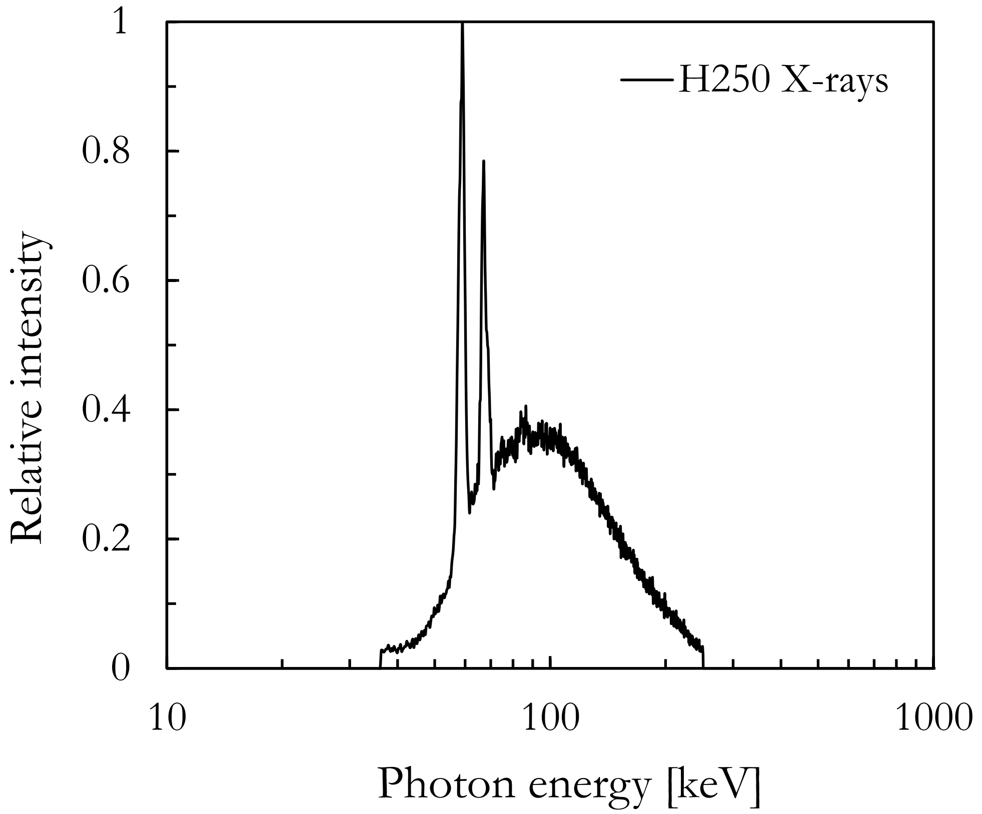

Figure SM1. Normalized energy spectrum of the H250 X-rays used for the cell exposures at SCK CEN, Mol, Belgium.

SM2. Radiation transport computer simulations

Radiation transport simulations were performed using the Particle and Heavy Ion Transport code System (PHITS, Sato et al 2018) version 3.28. A simplified geometry of the cell holder and of the material between the radiation source (i.e. the exit of the nozzle) and the cell holders (i.e. build up slabs) was implemented in PHITS. The simulated holder was filled with liquid water representing the cells and the medium. The simulated holder was irradiated with monoenergetic beams (energy listed in **Table 1** of the manuscript) impinging perpendicularly its front surface.

The following quantities were assessed in the first 100 μm of water within the holder (as representative of the position of the attached cells): dose-mean unrestricted linear energy transfer (LET) in water of the primary beam, dose-mean unrestricted LET in water of all particles (primary beam and all secondary particles), dose-mean lineal energy within homogenously distributed liquid water speres with diameter equal to 0.6 μm. This dimension was chosen as representative of the scale where the accumulation and repair of DNA damage correlates with cell death (Parisi et al., 2022).

The following physical models and options were used in the PHITS simulations:

- Macroscopic energy loss:
  - Charged particles (except of electrons and positrons): ATIMA.
  - Photons, electrons, and positrons: EGS5.
- Transport cutoffs: 1 keV/n (ions), 1 keV (all other particles but neutrons), 10^{-8} keV (neutrons).
- Angular straggling: Lynch's Coulomb diffusion formula based on Moliere theory.
- Energy straggling: Landau-Vavilov formula.
- Transport and interaction of low energy neutrons: event generator mode v2.
- Mean ionization potential of water = 78 eV.
- Nuclear reactions: default PHITS models.
- Microdosimetry: PHITS microdosimetric function.

More details can be found in Sato et al 2018, the PHITS manual (https://phits.jaea.go.jp/manual/manualE-phits.pdf), and references therein.

SM3. Tables of regression model outputs

Supplementary Table 1. Significant estimates for the expression of IL-1RA.

variable	coefficients	standard error	t-value	p-value
x_{cort}	-0.33	0.13	-2.6	0.009
$x_{smg} * x_{cort} * x_{d1}$	-0.5	0.24	-2.1	0.036
$x_{smg} * x_{Fe} * x_{d1}$	-0.76	0.23	-3.3	0.001
$x_{cort} * x_{C} * x_{d0.1}$	-0.74	0.24	-2.9	0.003
$x_{cort} * x_{Fe} * x_{d1}$	-0.59	0.24	-2.5	0.013
$x_{smg} * x_{cort} * x_{Fe} * x_{d1}$	0.99	0.33	3	0.003

Supplementary Table 2. Significant estimates for the expression of IL-6.

variable	coefficients	standard error	t-value	p-value
x_{cort}	-0.62	0.23	-2.7	0.007
$x_{smg} * x_{C}$	0.99	0.33	3	0.003
$x_{cort} * x_{smg} * x_{C}$	-0.96	0.46	-2.1	0.038
$x_{cort} * x_{smg} * x_{Fe} * x_{d1}$	2.04	0.68	2.9	0.003

Supplementary Table 3. Significant estimates for the expression of PDGF-α.

variable	coefficients	standard error	t-value	p-value
$x_{C} * x_{smg}$	4.41	0.75	5.9	< 0.0001
$x_{C} * x_{smg} * x_{cort}$	-4.39	1.51	-2.9	0.0042

Supplementary Table 4. Significant estimates for the expression of TGF-β.

variable	coefficients	standard error	t-value	p-value
x_{smg}	0.36	0.16	2.3	0.0209
$x_{smg} * x_{cort}$	-1.17	0.22	-5.3	< 0.0001
$x_{smg} * x_{Fe}$	-0.035	0.16	-2.2	0.0279
$x_{smg} * x_{cort} * x_{prot}$	0.55	0.23	2.4	0.0175
$x_{smg} * x_{cort} * x_{C}$	0.79	0.23	3.4	0.0007
$x_{smg} * x_{cort} * x_{Fe}$	0.97	0.23	4.3	< 0.0001
$x_{smg} * x_{cort} * x_{d1}$	0.55	0.22	2.5	0.0118

Supplementary Table 5. Coefficients of significant estimates for fibroblast migration.

variable	coefficients	standard error	t-value	p-value
x_{smg}	-0.24	0.03	-7.76	< 0.0001
x_{cort}	-0.06	0.03	-2.10	0.0368
$x_{smg} * x_{prot}$	0.11	0.04	2.85	0.0044
$x_{prot} * x_{cort}$	-0.07	0.04	-2.05	0.0405
$x_{cort} * x_{Fe}$	-0.19	0.04	-5.06	< 0.0001
$x_{prot} * x_{d1}$	-0.12	0.05	-2.25	0.0249

Supplementary Table 6. Coefficients of significant estimates for actin area after exposure to simulated spaceflight stressors.

variable	coefficients	standard error	t-value	p-value
x_{cort}	-0.37	0.09	-4.00	< 0.0001
$x_{d0.1}$	-0.32	0.09	-3.60	0.0004
$x_{d0.5}$	-0.19	0.09	-2.10	0.0356
x_{d1}	-0.26	0.09	-2.90	0.0044
$x_{cort} * x_{smg}$	0.28	0.13	2.20	0.0294
$x_{cort} * x_{d0.1}$	0.53	0.14	3.90	<0.0001
$x_{cort} * x_{d0.5}$	0.39	0.14	2.80	0.0047
$x_{cort} * x_{d1}$	0.32	0.13	2.40	0.0167
$x_{smg} * x_{d0.1}$	0.43	0.13	3.40	0.0008
$x_{cort} * x_{C}$	0.33	0.13	2.60	0.0105
$x_{cort} * x_{Fe}$	0.78	0.13	6.00	<0.0001
$x_{d0.1} * x_{Fe}$	0.44	0.13	3.40	0.0006
$x_{d0.5} * x_{Fe}$	0.28	0.13	2.20	0.0258
$x_{d1} * x_{Fe}$	0.62	0.13	4.70	<0.0001
$x_{cort} * x_{smg} * x_{d0.1}$	-0.77	0.19	-4.00	<0.0001
$x_{cort} * x_{smg} * x_{C}$	-0.41	0.19	-2.20	0.0315
$x_{cort} * x_{smg} * x_{Fe}$	-0.58	0.19	-3.10	0.0018
$x_{cort} * x_{d0.1} * x_{C}$	-0.43	0.19	-2.30	0.0244
$x_{cort} * x_{d0.1} * x_{Fe}$	-0.72	0.19	-3.80	0.0002
$x_{cort} * x_{d0.5} * x_{Fe}$	-0.60	0.19	-3.50	0.0016
$x_{cort} * x_{d1} * x_{Fe}$	-0.69	0.19	-3.70	0.0002
$x_{smg} * x_{d0.1} * x_{prot}$	-0.36	0.18	-2.00	0.0493
$x_{smg} * x_{d0.1} * x_{C}$	-0.70	0.19	-3.60	0.0003
$x_{smg} * x_{d0.1} * x_{Fe}$	-0.53	0.18	-2.90	0.0042
$x_{cort} * x_{smg} * x_{d0.1} * x_{prot}$	0.66	0.27	2.50	0.0137
$x_{cort} * x_{smg} * x_{d0.1} * x_{C}$	0.94	0.27	3.50	0.0005
$x_{cort} * x_{smg} * x_{d0.5} * x_{C}$	0.59	0.27	2.20	0.0302
$x_{cort} * x_{smg} * x_{d0.1} * x_{Fe}$	1.04	0.27	3.90	0.0001
$x_{cort} * x_{smg} * x_{d0.5} * x_{Fe}$	0.65	0.27	2.40	0.0147

Supplementary Table 7. Coefficients of significant estimates for number of stress fibers per cell.

variable	coefficients	standard error	t-value	p-value
actin area	1.63E-04	4.10E-06	39.7	< 0.0001
x_{d1}	-0.17	0.08	-2.10	0.0336
x_{prot}	0.16	0.08	2.00	0.0478
x_{C}	0.16	0.08	1.90	0.0519
x_{Fe}	0.20	0.08	2.50	0.0129
x_{cort}	-0.18	0.05	-3.60	0.0004
$x_{d1} * x_{C}$	0.27	0.11	2.60	0.0106
$x_{d1} * x_{Fe}$	0.26	0.11	2.40	0.0188
$x_{smg} * x_{d0.1}$	-0.31	0.11	-2.80	0.0060
$x_{cort} * x_{d0.5}$	0.15	0.06	2.80	0.0052
$x_{cort} * x_{Fe}$	0.40	0.06	7.10	<0.0001
$x_{smg} * x_{d0.1} * x_{C}$	0.48	0.16	3.10	0.0023
$x_{smg} * x_{d1} * x_{Fe}$	-0.39	0.15	-2.50	0.0111

Supplementary Table 8. Coefficients of significant estimates for the number of vinculin spots.

variable	coefficients	standard error	t-value	p-value
actin area	1.7 E-04	0.00	69.6	< 0.0001
x_{cort}	0.28	0.05	5.6	< 0.0001
x_{prot}	0.28	0.06	4.7	< 0.0001
x_{C}	0.23	0.06	3.9	0.0001
x_{Fe}	0.21	0.06	3.6	0.0004
x_{d1}	0.18	0.06	3.1	0.0021
$x_{cort} * x_{smg}$	-0.12	0.03	-4.8	< 0.0001
$x_{smg} * x_{d0.1}$	-0.16	0.07	-2.3	0.022
$x_{smg} * x_{d1}$	-0.18	0.07	-2.7	0.0075
$x_{prot} * x_{d1}$	-0.19	0.09	-2.1	0.027
$x_{C} * x_{d1}$	-0.17	0.08	-2.1	0.0357
$x_{Fe} * x_{d1}$	-0.19	0.09	-2.3	0.0228
$x_{cort} * x_{prot}$	-0.24	0.07	-3.4	0.0007
$x_{cort} * x_{C}$	-0.24	0.07	-3.4	0.0008
$x_{cort} * x_{Fe}$	-0.30	0.07	-4.4	< 0.0001
$x_{cort} * x_{d0.1}$	-0.17	0.07	-2.4	0.0169
$x_{smg} * x_{C} * x_{d1}$	0.29	0.10	3	0.0032
$x_{cort} * x_{C} * x_{d0.1}$	0.24	0.10	2.4	0.0186
$x_{cort} * x_{Fe} * x_{d0.1}$	0.38	0.10	3.8	0.0001
$x_{cort} * x_{Fe} * x_{d0.5}$	0.29	0.10	2.9	0.0035
$x_{cort} * x_{Fe} * x_{d1}$	0.38	0.10	3.9	0.0001

Supplementary Table 9. Coefficients of significant estimates for the synthesis of fibronectin.

variable	coefficients	standard error	t-value	p-value
x_{cort}	0.85	0.17	4.8	< 0.0001
x_{prot}	0.43	0.13	3.4	0.0008
x_C	0.29	0.13	2.2	0.0262
$x_{cort} * x_{d0.5}$	-0.46	0.21	-2.2	0.0309
$x_{cort} * x_{d1}$	-0.55	0.21	-2.6	0.0092
$x_{cort} * x_{Fe}$	-0.32	0.15	-2.2	0.03
$x_{smg} * x_{Fe}$	-0.41	0.15	-2.7	0.0063
$x_{cort} * x_{smg} * x_{d0.5}$	0.88	0.29	3	0.0032
$x_{cort} * x_{smg} * x_{d1}$	1.35	0.3	4.5	<0.0001

Table 10. Coefficients of significant estimates for the expression of type I $\alpha1$ procollagen.

variable	coefficients	standard error	t-value	p-value
x_{Fe}	0.48	0.17	2.9	0.0044
x_{smg}	-0.48	0.15	-3.1	0.0020
$x_{smg} * x_{cort}$	0.37	0.15	2.4	0.0152
$x_{cort} * x_{Fe}$	0.90	0.20	4.5	< 0.0001
$x_{smg} * x_{Fe}$	0.47	0.20	2.4	0.0181

Supplementary Table 11. Coefficients of significant estimates for the expression of type I $\alpha2$ procollagen.

variable	coefficients	standard error	t-value	p-value
x_{prot}	0.31	0.12	2.6	0.0096
x_C	-0.29	0.14	-2.2	0.035
x_{Fe}	0.23	0.11	2.1	0.0363
x_{smg}	-0.21	0.06	-3.43	0.0007
$x_{cort} * x_{prot}$	-0.37	0.17	-2.2	0.0282

References

- Parisi, A., Beltran, C.J. and Furutani, K.M., 2022. The Mayo Clinic Florida microdosimetric kinetic model of clonogenic survival: formalism and first benchmark against in vitro and in silico data. Physics in Medicine & Biology, 67(18), p.185013.
- Sato, T., Iwamoto, Y., Hashimoto, S., Ogawa, T., Furuta, T., Abe, S.I., Kai, T., Tsai, P.E., Matsuda, N., Iwase, H. and Shigyo, N., 2018. Features of particle and heavy ion transport code system (PHITS) version 3.02. Journal of Nuclear Science and Technology, 55(6), pp.684-690.
